# Tumoral RCOR2 promotes tumor development through dual epigenetic regulation of tumor plasticity and immunogenicity

**DOI:** 10.1172/JCI188801

**Published:** 2025-07-03

**Authors:** Lei Bao, Ming Zhu, Maowu Luo, Ashwani Kumar, Yan Peng, Chao Xing, Yingfei Wang, Weibo Luo

**Affiliations:** 1 Department of Pathology,; 2 Eugene McDermott Center for Human Growth and Development,; 3 Lyda Hill Department of Bioinformatics,; 4 O’Donnell School of Public Health,; 5 Department of Neurology,; 6 Peter O’Donnell Jr. Brain Institute, and; 7 Department of Pharmacology, University of Texas (UT) Southwestern Medical Center, Dallas, Texas, USA.

**Keywords:** Immunology, Oncology, Adult stem cells, Cancer immunotherapy, MHC class 2

## Abstract

Gain of plasticity and loss of MHC-II enable tumor cells to evade immune surveillance, contributing to tumor development. Here, we showed that the transcriptional corepressor RCOR2 is a key factor that integrates two epigenetic programs surveilling tumor plasticity and immunogenicity. RCOR2 was upregulated predominantly in tumor cells and promoted tumor development in mice through reducing tumor cell death by CD4^+^CD8^+^ T cells and inducing cancer stemness. Mechanistically, RCOR2 repressed RNF43 expression through LSD1-mediated demethylation of histone H3 at lysine 4 to induce activation of Wnt/β-catenin and tumor stemness. Simultaneously, RCOR2 inhibited CIITA expression through HDAC1/2-mediated deacetylation of histone H4 at lysine 16, leading to MHC-II silencing in tumor cells and subsequent impairment of CD4^+^CD8^+^ T cell immunosurveillance, thereby promoting immune evasion. RCOR2 loss potentiated anti–PD-1 therapy in mouse models of cancer and correlated with better response to anti–PD-1 therapy in human patients. Collectively, these findings uncover a “two birds with one stone” effect for RCOR2, highlighting its potential as a valuable target for improved cancer therapy.

## Abstract 2

One transcriptional complex for two distinct cancer phenotypes

## Graphical abstract

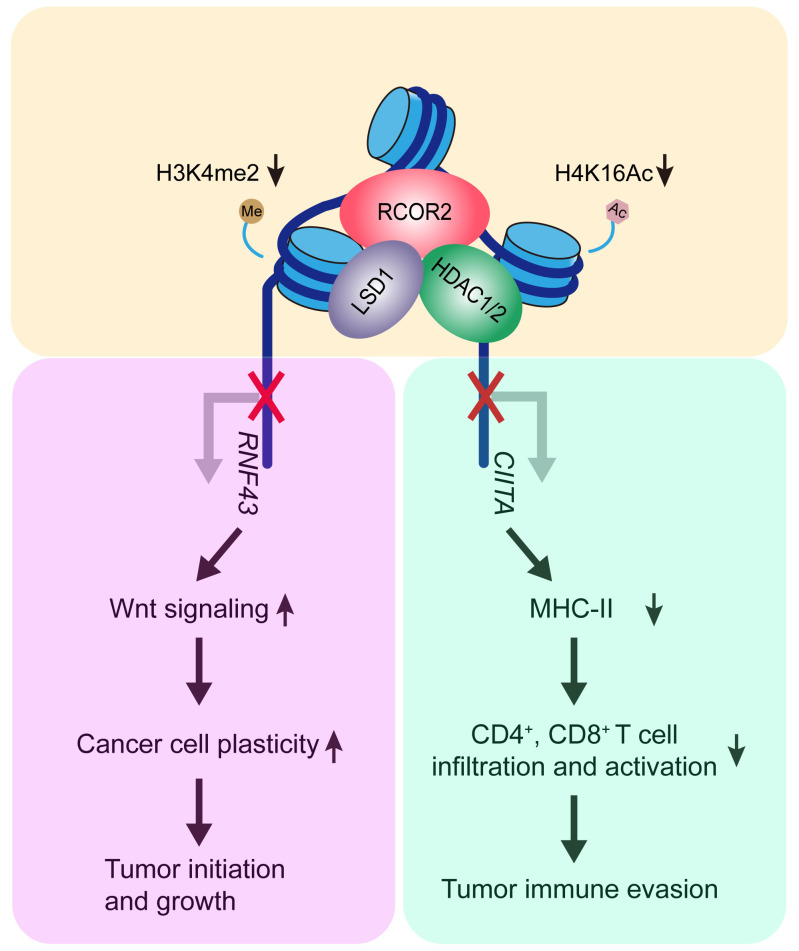

## Introduction

Human tumor cells often escape from immune attack, leading to tumor initiation, progression, and recurrence. Dysfunction in antigen presentation, a key process in the course of immune defense, contributes to immune evasion and resistance to immune checkpoint blockade (ICB) therapies (1–3). Tumor antigens are processed and presented by major histocompatibility complex (MHC) molecules, consisting of MHC class I (MHC-I) and class II (MHC-II) (4). Subsequently, T cell receptors can recognize tumor antigen–MHC complexes, leading to activation and expansion of cytotoxic CD8^+^ and CD4^+^ T cells, respectively (5). Numerous efforts have focused on harnessing MHC-I–mediated CD8^+^ T cell activation for immunotherapies in human cancers (3, 6–9). In contrast to their well-established role in assisting CD8^+^ T cell activation (10), the direct function of CD4^+^ T cells as effector cells in antitumor immunity is less studied (11), although several reports have shown that a subpopulation of CD4^+^ T cells exhibits cytotoxicity against tumor cells with high levels of MHC-II (12–16). Unlike ubiquitously expressed MHC-I, MHC-II molecules are predominantly expressed in professional antigen-presenting cells (APCs), including dendritic cells, B cells, and macrophages, which are induced by the class II transactivator (CIITA) and interferon-γ (IFN-γ) (17–19). Emerging studies have revealed that tumor cells can act as APCs and heterogeneously express MHC-II (20–22). Tumoral MHC-II expression positively correlates with a superior prognosis and improved response to ICB treatment (23–26), suggesting that inducing tumoral MHC-II expression may boost antitumor immunity in human cancers. However, MHC-II molecules are frequently downregulated in most tumor cells (27), which raises a fundamental question of whether MHC-II can be induced to increase tumor immunogenicity and achieve durable and robust antitumor immunity in human cancers.

Altered tumor cell behaviors are also critical factors that determine the tumor’s response to immune surveillance (28). Tumor cells are highly plastic and acquire remarkable intrinsic adaptability to sustain their plasticity. Various signaling pathways, including Wnt/β-catenin, Notch, and Hedgehog, are activated in response to intrinsic and extrinsic stimuli in tumor cells and contribute to maintenance of cancer stemness (29, 30). These plastic characteristics enable tumor cells to develop resistance to immunotherapies. For example, activation of epithelial-mesenchymal transition confers immunosuppression in the tumor microenvironment and resistance of tumor cells to ICB therapies (31). However, the mechanism by which tumor cells jointly orchestrate the plastic phenotype alongside immune evasion to promote tumor development and treatment resistance, and whether these processes are coregulated, remain largely unknown.

RCOR2 belongs to the evolutionarily conserved CoREST family, consisting of 3 members, RCOR1–3 (32). As a scaffold protein, RCOR2 binds 2 distinct catalytic subunits, including histone deacetylases HDAC1 and HDAC2 through its N-terminal ELM2 and SANT1 domains and histone demethylase LSD1 through its C-terminal SANT2 domain, as well as other subunits to form a transcriptional corepressor complex, which induces silencing of genes whose protein products are involved in cell differentiation, stem cell pluripotency, and neurogenesis (32–34). Although RCOR1–3 bind to the same key complex components, structural studies showed that the RCOR2 complex has a distinct conformation compared with RCOR1 and RCOR3 complexes (35), suggesting that the RCOR2 complex may have a unique role in the regulation of gene repression.

In this study, we showed that RCOR2 was upregulated predominantly in tumor cells and promoted tumor development by simultaneously increasing tumor cell plasticity and immune evasion. RCOR2 hijacked LSD1- and HDAC1/2-dependent epigenetic programs to promote tumor plasticity and immune evasion, respectively. Targeting RCOR2 potentiated ICB therapy in mouse models of cancer. Collectively, these findings uncover a potential therapeutic target and biomarker for cancer prognosis and treatment.

## Results

### RCOR2 is upregulated primarily in tumor cells across human cancers.

**Figure 1:**
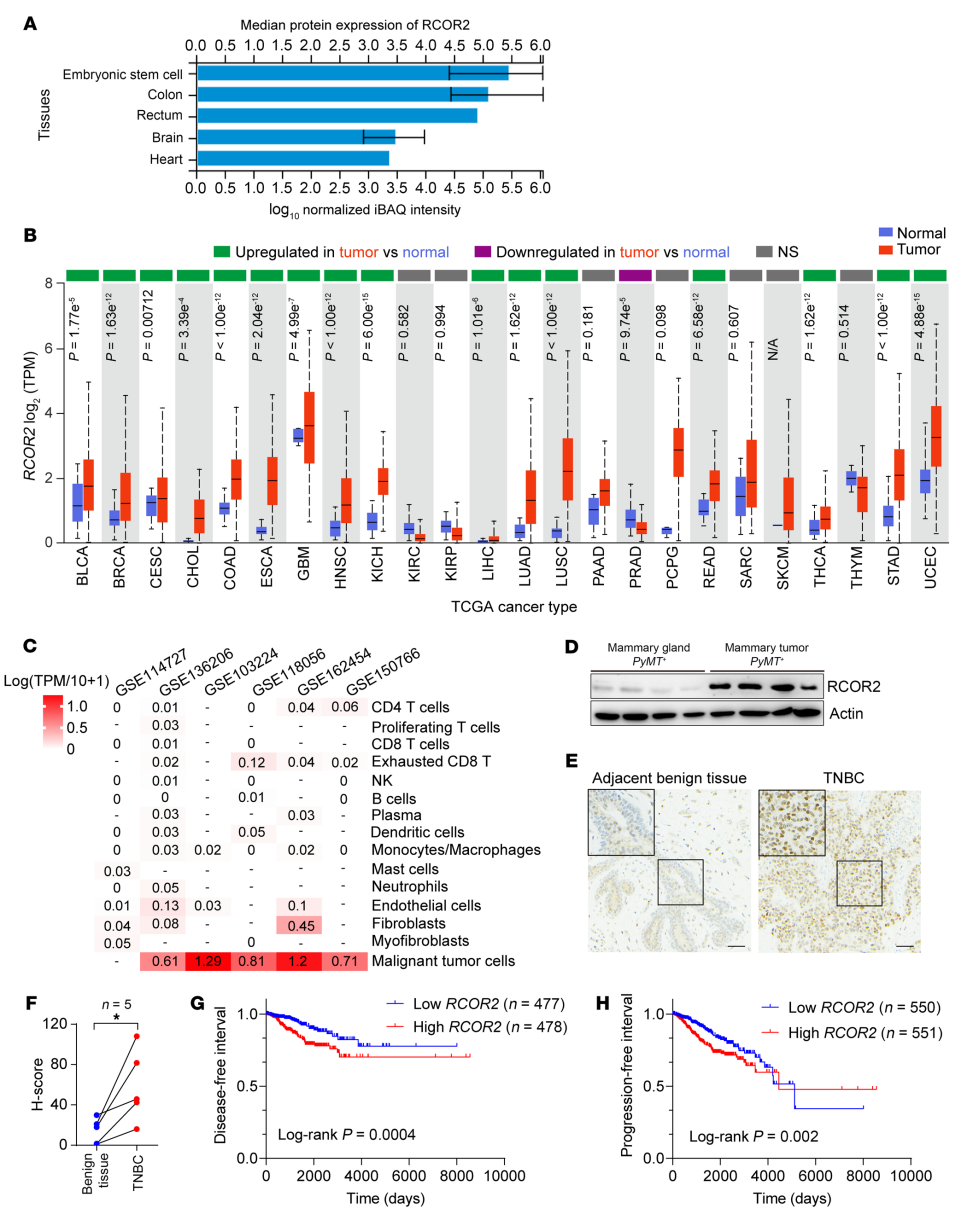
RCOR2 is upregulated in cancer cells and predicts poor survival in breast cancer patients. (**A**) Mass spectrometry analysis of RCOR2 protein levels in human tissues. Data were retrieved from ProteomicsDB. (**B**) mRNA expression analysis of *RCOR2* across various types of human tumors and normal tissues from TCGA. *P* values were calculated by unequal-variance *t* test. Data were retrieved from UALCAN. N/A, not applicable; NS, not significant. (**C**) Single-cell RNA-Seq analysis of *RCOR2* in tumors. Data were retrieved from TISCH2. (**D**) Immunoblot analysis of RCOR2 and actin proteins in normal mammary gland and *MMTV-PyMT* mammary tumors from mice. (**E** and **F**) Representative RCOR2 IHC in human triple-negative breast tumors and adjacent benign tissues (**E**); staining is quantified with H-score (**F**). *P < 0.05 by paired 2-tailed Student’s *t* test. Scale bars: 50 μm. (**G** and **H**) Kaplan-Meier survival analysis for patients with breast cancer by log-rank test. Patients were divided by median expression levels of *RCOR2* mRNA. Data were retrieved from TCGA. iBAQ, intensity-based absolute quantification.

The proteomic analysis of human tissues revealed that RCOR2 expression was restricted to embryonic stem cells and a few human adult tissues, including colon, rectum, brain, and heart (Figure 1A). Intriguingly, we found widespread upregulation of *RCOR2* mRNA in various types of human cancers in The Cancer Genome Atlas (TCGA) cohort (Figure 1B), predominantly expressed in malignant tumor cells (Figure 1C). RCOR2 protein upregulation was confirmed in murine MMTV-PyMT mammary tumors and human triple-negative breast cancer (Figure 1, D–F). Kaplan-Meier analysis of the TCGA cohort revealed that high levels of *RCOR2* were significantly associated with worse disease-free interval and progression-free interval in breast cancer patients (Figure 1, G and H). These findings indicate that RCOR2 expression is awakened in tumors and may play a critical role in cancer development.

### Tumoral RCOR2 inhibits cytotoxic T cell infiltration to promote tumor growth in mice.

**Figure 2:**
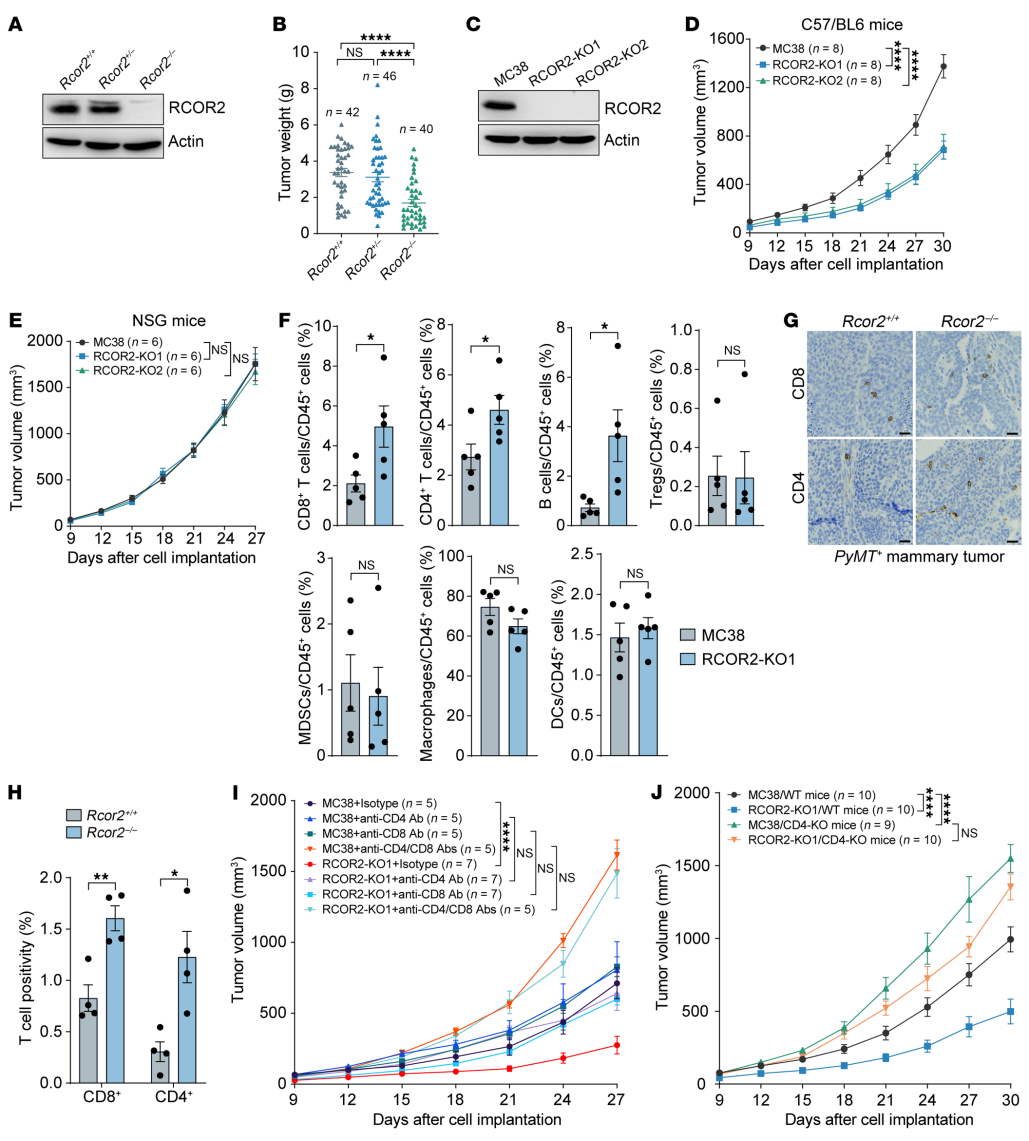
RCOR2 promotes tumor immune evasion in mice. (**A** and **B**) Immunoblot (**A**) and weight (**B**) of mammary tumors in *MMTV-PyMT^+/–^* *K14-Cre^+/–^* *Rcor2^+/+^*, *MMTV-PyMT^+/–^* *K14-Cre^+/–^* *Rcor2^+/fl^*, and *MMTV-PyMT^+/–^* *K14-Cre^+/–^* *Rcor2^fl/fl^* mice. (**C**) Immunoblot analysis of RCOR2 protein in parental and RCOR2-KO1 or -KO2 MC38 cells. (**D** and **E**) Growth of parental and RCOR2-KO1 or -KO2 MC38 tumors in C57BL/6J (**D**) and NSG (**E**) mice. (**F**) Flow cytometry analysis of CD8^+^ T cells (CD45^+^CD3e^+^CD8^+^), CD4^+^ T cells (CD45^+^CD3e^+^CD4^+^), B cells (CD45^+^B220^+^), regulatory T cells (CD45^+^CD3e^+^CD4^+^FOXP3^+^), myeloid-derived suppressor cells (MDSCs; CD45^+^CD11b^+^Gr-1^+^), macrophages (CD45^+^CD11b^+^F4/80^+^), and dendritic cells (CD45^+^CD11c^+^F4/80^–^) in parental and RCOR2-KO1 MC38 tumors (*n* = 5). (**G** and **H**) CD8 and CD4 IHC in *MMTV-PyMT^+/–^* *K14-Cre^+/–^* *Rcor2^+/+^* and *MMTV-PyMT^+/–^* *K14-Cre^+/–^* *Rcor2^fl/fl^* tumors (**G**); the percentage of T cells is quantified (**H**) (*n* = 4). Scale bars: 25 μm. (**I**) Growth of parental and RCOR2-KO1 MC38 tumors in C57BL/6J mice treated with IgG or anti-CD4 and anti-CD8 neutralizing antibodies. (**J**) Growth of parental and RCOR2-KO1 MC38 tumors in C57BL/6J and CD4-KO mice. Data represent mean ± SEM. *P* values were determined by 1-way ANOVA with Tukey’s test (**B**), 2-way ANOVA with Tukey’s test (**I** and **J**) or Dunnett’s test (**D** and **E**), and 2-tailed Student’s *t* test (**F** and **H**). *P < 0.05; **P < 0.01; ****P < 0.0001.

To determine a role of RCOR2 in tumor progression, we crossed *Rcor2*-floxed mice with K14-Cre and MMTV-PyMT transgenic mice and monitored mammary tumor growth in mice over 5 months. RCOR2 protein was depleted by K14-Cre in PyMT tumors harvested from homozygous *Rcor2*-floxed mice but not wild-type and heterozygous mice (Figure 2A). Homozygous deletion of *Rcor2* significantly inhibited PyMT mammary tumor growth in mice (Figure 2B). To validate the results from the genetically modified mammary tumor mouse model, we conducted allograft experiments by implanting parental and RCOR2-knockout (KO) murine tumor cells into the mammary fat pad of female BALB/c mice or the flank of male C57BL/6J mice. RCOR2 KO significantly inhibited growth of MC38 colorectal tumors and TUBO mammary tumors in the syngeneic mouse models (Figure 2, C and D, and Supplemental Figure 1, A and B; supplemental material available online with this article; https://doi.org/10.1172/JCI188801DS1). To our surprise, the inhibitory role of RCOR2 KO in murine tumor growth was abolished in immunodeficient NSG mice (Figure 2E and Supplemental Figure 1C). We further confirmed that RCOR2 KO1 or KO2 did not inhibit human tumor growth in NSG mice orthotopically implanted with 2 million human MDA-MB-231 breast cancer cells (Supplemental Figure 1, D–H). Consistently, RCOR2 KO1 or KO2 had no effect on breast cancer cell proliferation and colony growth in vitro (Supplemental Figure 1, I–K). These results indicate that RCOR2 promotes tumor growth in a manner that relies on the host’s immune system.

We next conducted immune cell profiling by flow cytometry to comprehensively assess the effect of tumoral RCOR2 on the composition and abundance of immune cell subsets within tumors (Supplemental Figure 2, A and B). The percentage of intratumoral lymphocytes including CD4^+^ T cells, CD8^+^ T cells, and B cells was significantly increased in RCOR2-KO1 MC38 tumors as compared with their control tumors (Figure 2F). A similar effect on CD4^+^ and CD8^+^ T cell infiltration was observed in RCOR2-KO1 TUBO tumors (Supplemental Figure 2C). Immunohistochemistry (IHC) analysis further confirmed increased infiltration of CD4^+^ and CD8^+^ T cells in RCOR2-KO PyMT mammary tumors compared with wild-type tumors (Figure 2, G and H). In contrast, RCOR2 KO1 had no effect on infiltration of regulatory T cells and myeloid cells including myeloid-derived suppressor cells, macrophages, and dendritic cells in MC38 and TUBO tumors (Figure 2F and Supplemental Figure 2C). These results indicate that tumoral RCOR2 shapes the lymphocyte landscape in the tumor microenvironment.

To determine whether loss of CD4^+^ and/or CD8^+^ T cells is necessary for RCOR2-mediated tumor growth, we administered anti-CD4 antibody, anti-CD8 antibody, anti-CD4/CD8 antibodies or control antibody isotype intraperitoneally into tumor-bearing mice to deplete CD4^+^ and CD8^+^ T cells. Depletion of CD4^+^ T cells, CD8^+^ T cells, or both with anti-CD4/CD8 neutralizing antibodies effectively restored RCOR2-KO1 tumors in the murine TUBO mammary tumor model (Supplemental Figure 2D). More robust rescue of RCOR2-KO1 tumors was observed in the MC38 tumor mouse model when mice were cotreated with anti-CD4/CD8 antibodies (Figure 2I). Notably, genetic deletion of CD4^+^ T cells greatly promoted MC38 tumor growth and abolished tumor reduction conferred by RCOR2 KO in mice (Figure 2J), supporting an inhibitory role of CD4^+^ T cells in RCOR2-induced tumor growth, either directly or indirectly through their regulation of other immune components. Collectively, these results indicate that RCOR2 promotes tumor growth through reducing infiltration of CD4^+^ and CD8^+^ T cells.

### RCOR2 increases intrinsic cancer cell plasticity to promote tumor development in mice.

**Figure 3:**
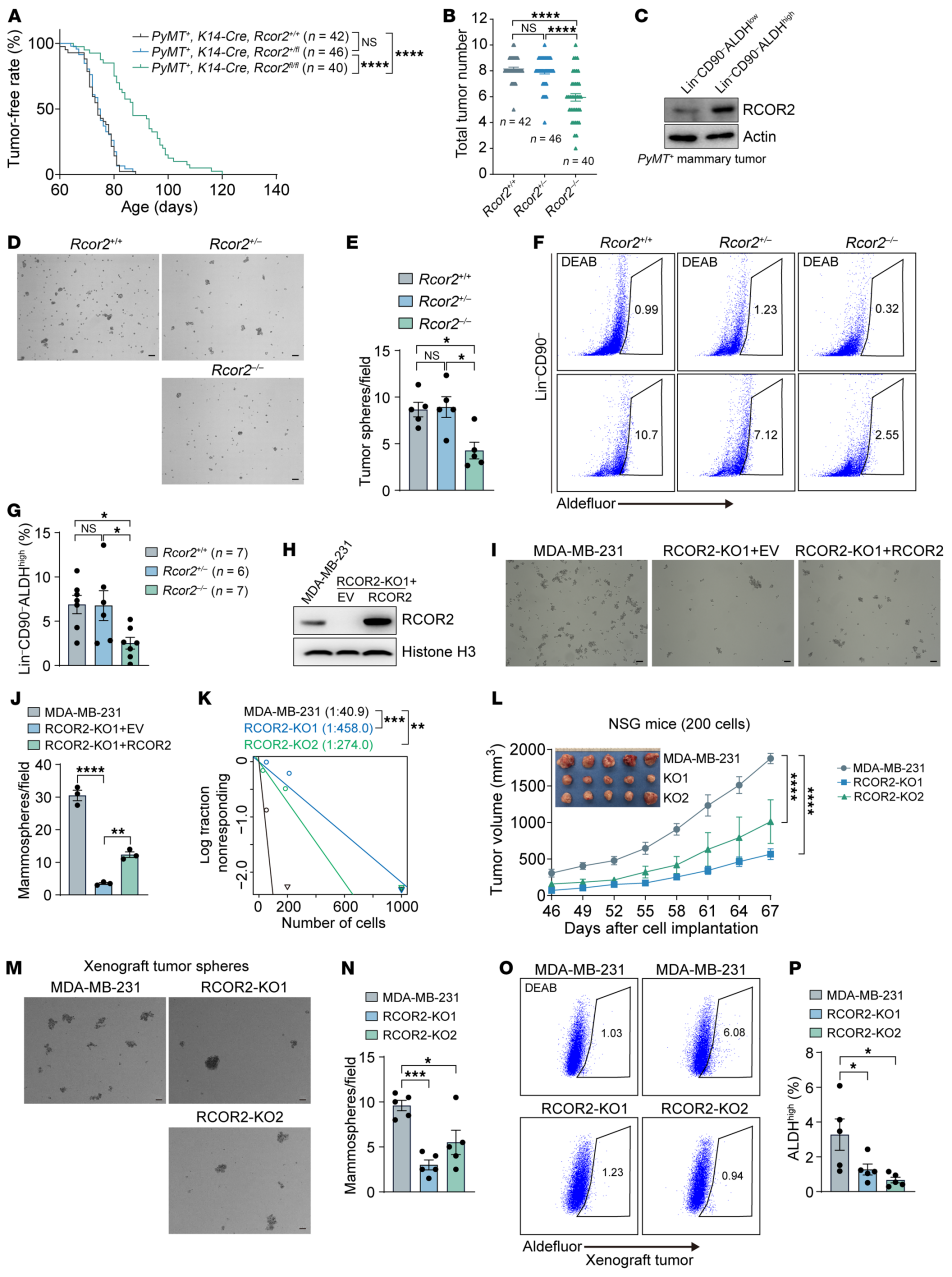
RCOR2 enhances tumor cell plasticity to promote cancer development. (**A** and **B**) Tumor-free period (**A**) and mammary tumor number (**B**) of *MMTV-PyMT^+/–^* *K14-Cre^+/–^* *Rcor2^+/+^*, *MMTV-PyMT^+/–^* *K14-Cre^+/–^* *Rcor2^+/fl^*, and *MMTV-PyMT^+/–^* *K14-Cre^+/–^* *Rcor2^fl/fl^* mice. (**C**) Immunoblot analysis of RCOR2 protein in non-tumor-initiating cells (Lin^–^CD90^–^ALDH^lo^) and tumor-initiating cells (Lin^–^CD90^–^ALDH^hi^) isolated from *MMTV-PyMT* tumors. (**D** and **E**) Tumorsphere formation assay of *MMTV-PyMT^+/–^* *K14-Cre^+/–^* *Rcor2^+/+^*, *MMTV-PyMT^+/–^* *K14-Cre^+/–^* *Rcor2^+/fl^*, and *MMTV-PyMT^+/–^* *K14-Cre^+/–^* *Rcor2^fl/fl^* tumors. Representative tumorsphere images are shown in **D**. Tumorsphere numbers are quantified in **E** (*n* = 5). (**F** and **G**) Flow cytometry analysis (**F**) and quantification (**G**) of tumor-initiation cells in *MMTV-PyMT^+/–^* *K14-Cre^+/–^* *Rcor2^+/+^*, *MMTV-PyMT^+/–^* *K14-Cre^+/–^* *Rcor2^+/fl^*, and *MMTV-PyMT^+/–^* *K14-Cre^+/–^* *Rcor2^fl/fl^* tumors. Representative gating is shown in **F**. ALDH^hi^ cells are quantified in **G**. (**H**) Immunoblot analysis of RCOR2 protein in parental, RCOR2-KO1, and RCOR2-rescue MDA-MB-231 cells. (**I** and **J**) Mammosphere formation assay of parental, RCOR2-KO1, and RCOR2-rescue MDA-MB-231 cells. Representative mammosphere images are shown in **I**. Mammosphere numbers are quantified in **J** (*n* = 3). (**K**) Limiting dilution assay of parental and RCOR2-KO1 or -KO2 MDA-MB-231 cells in NSG mice. (**L**) Growth of parental and RCOR2-KO1 or -KO2 MDA-MB-231 tumors in NSG mice. (**M** and **N**) Tumorsphere formation assay in parental and RCOR2-KO1 or -KO2 MDA-MB-231 tumors. Representative tumorsphere images are shown in **M**. Tumorsphere numbers are quantified in **N** (*n* = 5). (**O** and **P**) Aldefluor assay (STEMCELL Technologies) in parental and RCOR2-KO1 or -KO2 MDA-MB-231 tumors. Representative flow cytometry gating is shown in **O**. ALDH^hi^ cells are quantified in **P** (*n* = 5). Data represent mean ± SEM. *P* values were determined by 1-way ANOVA with Tukey’s test (**B**, **E**, **G**, and **J**) or Dunnett’s test (**N** and **P**), 2-way ANOVA with Dunnett’s test (**L**), log-rank (Mantel-Cox) test (**A**), and χ^2^ test (**K**). *P < 0.05; **P < 0.01; ***P < 0.001; ****P < 0.0001. Scale bars: 100 μm (**D**, **I**, and **M**).

Homozygous deletion of *Rcor2* significantly decreased incidence and numbers of murine PyMT tumors in mice (Figure 3, A and B), indicating that RCOR2 promotes tumor initiation. To determine whether RCOR2 controls cancer cell plasticity leading to tumor initiation, we isolated aldehyde dehydrogenase–high (ALDH^hi^) breast cancer stem cells (BCSCs; Lin^–^CD90^–^ALDH^hi^) from PyMT mammary tumors by flow cytometry and found elevated RCOR2 protein in this cell population compared with Lin^–^CD90^–^ALDH^lo^ non-BCSCs (Figure 3C). Loss of RCOR2 blocked PyMT tumorsphere formation ex vivo and reduced ALDH^hi^ BCSCs in PyMT tumors in vivo (Figure 3, D–G).

To validate the role of RCOR2 in cancer cell plasticity observed in a murine mammary tumor model, we generated BCSC-enriched mammospheres from human breast cancer cells. In line with murine tumors, RCOR2 protein levels were remarkably increased in MDA-MB-231 and MCF-7 mammospheres compared with their monolayers with scarcely detectable BCSCs (Supplemental Figure 3, A and B). Forced expression of RCOR2 significantly increased formation of MDA-MB-231 mammospheres (Supplemental Figure 3, C–E). In contrast, RCOR2 KO1 or KO2 decreased the number of MDA-MB-231 and MCF-7 mammospheres (Supplemental Figure 3, F–J). The RCOR2 loss-of-function effect was specific, as re-expression of RCOR2 could partially restore formation of RCOR2-KO1 mammospheres (Figure 3, H–J). We further showed that RCOR2 KO1 or KO2 significantly decreased the proportion of ALDH^hi^ BCSCs in MDA-MB-231 and MCF-7 cells as well as in MDA-MB-231 mammospheres (Supplemental Figure 3, K–P). CD44^+^CD24^–^EpCAM^+^ BCSC populations were also decreased by RCOR2 loss in MDA-MB-231 and MCF-7 cells (Supplemental Figure 3, Q and R). Collectively, these results indicate that RCOR2 is strongly expressed in ALDH^hi^ BCSCs and is sufficient and necessary for cancer cell plasticity.

To determine whether RCOR2 controls tumor cell plasticity to promote tumor development, we performed limiting dilution assay in NSG mice. Parental and RCOR2-KO1 or -KO2 MDA-MB-231 cells with 3 cell numbers of 40, 200, and 1,000 were orthotopically implanted into the mammary fat pad of female NSG mice. RCOR2 KO1 or KO2 significantly decreased the tumor incidence in NSG mice (Figure 3K). Similar results were observed in the MCF-7 xenograft mouse models (Supplemental Figure 3S). Notably, RCOR2 KO significantly inhibited MDA-MB-231 tumor growth in NSG mice when a limited number of cancer cells were implanted (Figure 3L). We confirmed reduced ALDH^hi^ BCSCs within tumors and ex vivo tumorsphere formation by RCOR2 KO1 or KO2 (Figure 3, M–P). Collectively, these results indicate that RCOR2 enhances cancer cell plasticity to promote tumor development.

### RCOR2 activates Wnt/β-catenin signaling but suppresses CIITA/MHC-II signaling in cancer cells through two distinct epigenetic programs.

**Figure 4:**
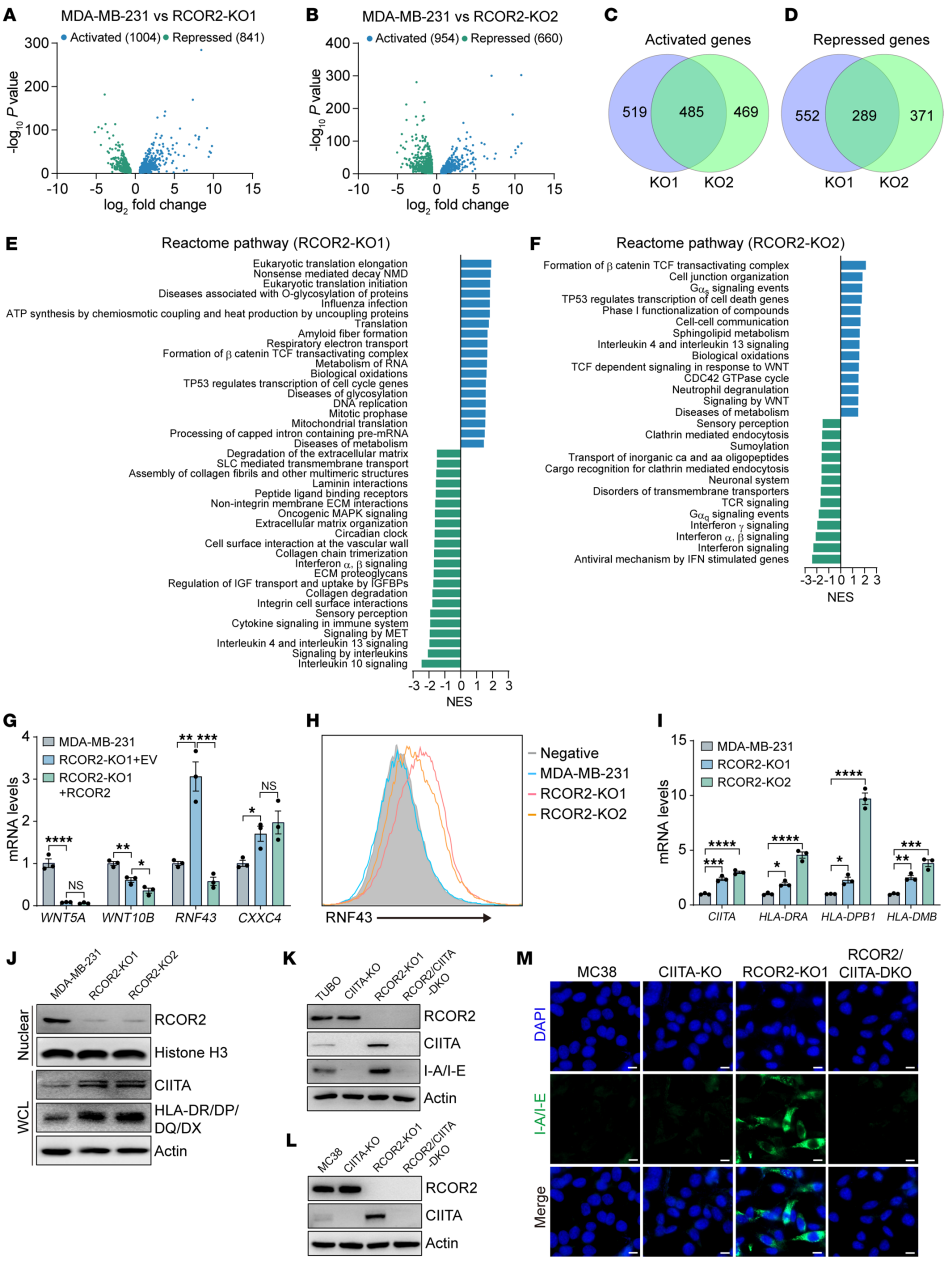
RCOR2 activates Wnt/β-catenin by repressing RNF43 and inhibits immune response by repressing CIITA and MHC-II. (**A** and **B**) Volcano plots of RCOR2 target genes in MDA-MB-231 cells (*n* = 2). (**C** and **D**) Venn diagrams of RCOR2 activated (**C**) and repressed (**D**) gene numbers in MDA-MB-231 cells (*n* = 2). (**E** and **F**) Reactome pathway analysis of RCOR2 target genes in MDA-MB-231 cells (*n* = 2). (**G**) RT-qPCR analysis of indicated mRNAs in parental, RCOR2-KO, and RCOR2-rescue MDA-MB-231 cells (*n* = 3). (**H**) Flow cytometry analysis of RNF43 protein in parental and RCOR2-KO1 or -KO2 MDA-MB-231 cells. (**I**) RT-qPCR analysis of indicated mRNAs in parental and RCOR2-KO1 or -KO2 MDA-MB-231 cells treated with 0.1 ng/mL IFN-γ for 24 hours (*n* = 3). (**J**) Immunoblot analysis of indicated proteins in parental and RCOR2-KO1 or -KO2 MDA-MB-231 cells treated with 0.1 ng/mL IFN-γ for 24 hours. (**K**) Immunoblot analysis of indicated proteins in parental, RCOR2-KO1, CIITA-KO, and RCOR2/CIITA–DKO TUBO cells treated with 1 ng/mL IFN-γ for 24 hours. (**L**) Immunoblot analysis of indicated proteins in parental, RCOR2-KO1, CIITA-KO, and RCOR2/CIITA–DKO MC38 cells treated with 5 ng/mL IFN-γ for 24 hours. (**M**) Representative immunostaining of I-A/I-E in parental, RCOR2-KO1, CIITA-KO, and RCOR2/CIITA–DKO MC38 cells treated with 5 ng/mL IFN-γ for 24 hours. Scale bars: 10 μm. Data represent mean ± SEM. *P* values were determined by 1-way ANOVA with Tukey’s test (**G**) or Dunnett’s test (**I**). *P < 0.05; **P < 0.01; ***P < 0.001; ****P < 0.0001.

To determine the mechanism by which tumoral RCOR2 promotes tumor cell plasticity and immune evasion, we assessed RCOR2 transcriptome in MDA-MB-231 cells by RNA sequencing (RNA-Seq). Four hundred eighty-five genes were induced whereas 289 genes were repressed by RCOR2 (FDR < 0.05; log counts per million > 0; |fold change| > 1.5; Figure 4, A–D). Reactome pathway analysis of these differentially expressed genes revealed that activation of the Wnt/β-catenin signaling pathway and inhibition of the interferon signaling pathway were shared in both RCOR2-KO1 and -KO2 cells (Figure 4, E and F). Reverse transcription–quantitative polymerase chain reaction (RT-qPCR) assay confirmed repression of two Wnt ligands, *WNT5A* and *WNT10B*, and induction of two negative regulators of the Wnt/β-catenin pathway, *RNF43* and *CXXC4*, in RCOR2-KO1 and -KO2 MDA-MB-231 cells (Supplemental Figure 4A). However, re-expression of RCOR2 caused derepression of *RNF43* only in RCOR2-KO cells (Figure 4G). RNF43 protein levels were also increased by RCOR2 loss in MDA-MB-231 cells and PyMT tumors (Figure 4H and Supplemental Figure 4, B and C). These results indicate that RCOR2 represses RNF43 expression in cancer cells.

By searching differentially expressed genes involved in interferon signaling pathways from our RNA-Seq dataset (Figure 4, A–D), we found that a family of MHC-II heavy chain genes and their transcriptional coactivator *CIITA* were repressed by RCOR2, which was validated in multiple cancer cell lines by RT-qPCR assay (Figure 4I and Supplemental Figure 4, D and E). Protein levels of CIITA and MHC-II molecules were also elevated in RCOR2-depleted cancer cells following IFN-γ treatment and in PyMT tumors, as shown by immunoblot, flow cytometry, and/or immunostaining assays (Figure 4J and Supplemental Figure 4, F–J). CIITA KO counteracted RCOR2 KO1–induced MHC-II molecules in TUBO and MC38 cells (Figure 4, K–M), suggesting that RCOR2 indirectly reduces MHC-II expression in tumor cells by repressing CIITA. We further found that RCOR2 had no effect on MHC-I expression in cancer cells (Figure 4, A and B, and Supplemental Figure 4E). Together, these findings indicate that RCOR2 specifically induces MHC-II silencing in cancer cells through suppression of CIITA.

**Figure 5:**
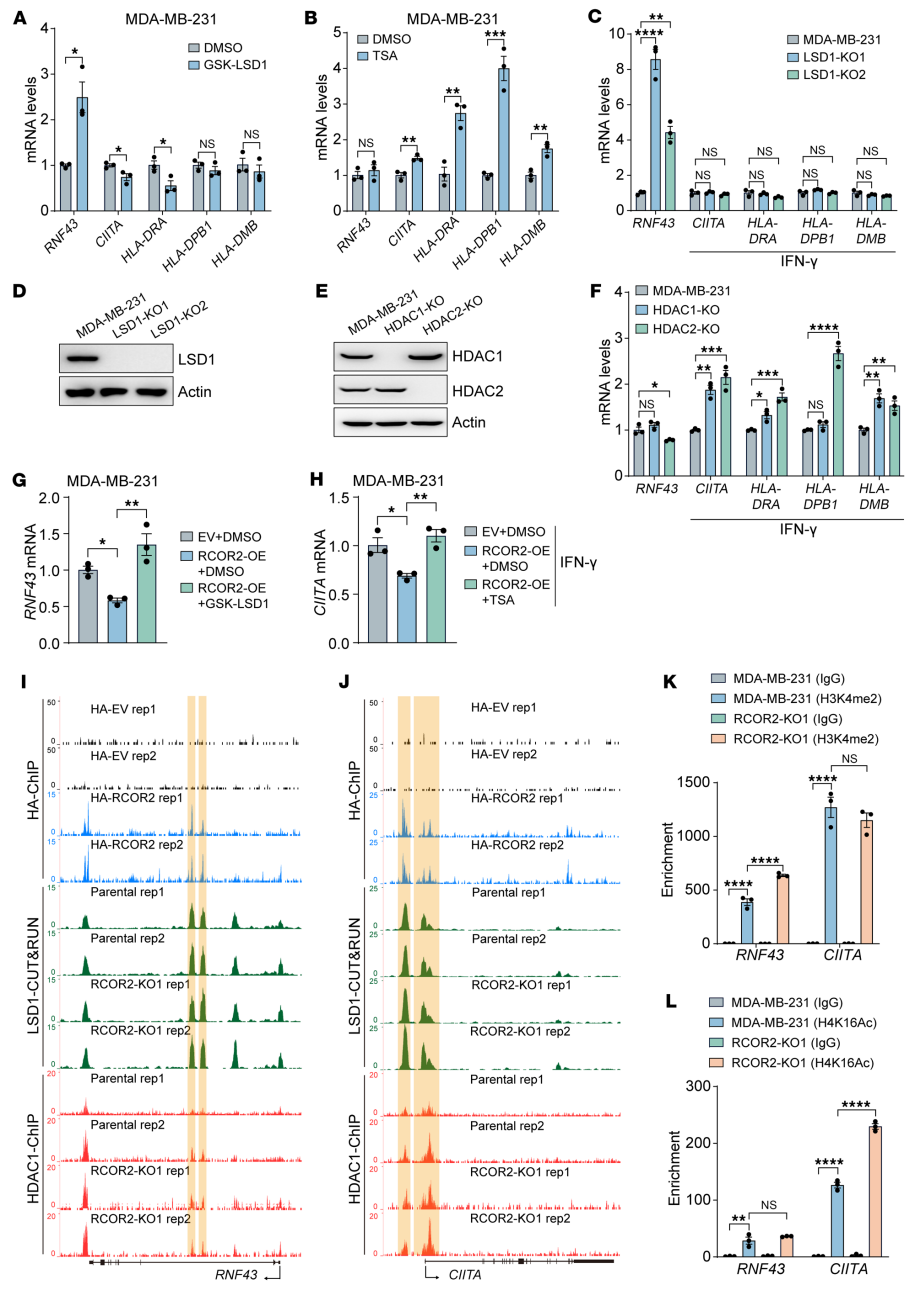
RCOR2 inhibits RNF43 and CIITA expression via distinct epigenetic mechanisms. (**A** and **B**) RT-qPCR analysis of indicated mRNAs in MDA-MB-231 cells treated with 50 μM GSK-LSD1 (**A**) or 0.2 μM TSA (**B**) for 48 hours (*n* = 3). (**C**) RT-qPCR analysis of indicated mRNAs in parental and LSD1-KO MDA-MB-231 cells treated with or without 0.1 ng/mL IFN-γ for 24 hours (*n* = 3). (**D**) Immunoblot analysis of indicated proteins in parental and LSD1-KO MDA-MB-231 cells. (**E**) Immunoblot analysis of indicated proteins in parental, HDAC1-KO, and HDAC2-KO MDA-MB-231 cells. (**F**) RT-qPCR analysis of indicated mRNAs in parental, HDAC1-KO, and HDAC2-KO MDA-MB-231 cells treated with or without 0.1 ng/mL IFN-γ for 24 hours (*n* = 3). (**G**) RT-qPCR analysis of indicated mRNAs in MDA-MB-231 cells overexpressing empty vector (EV) or RCOR2 treated with DMSO or 50 μM GSK-LSD1 for 48 hours (*n* = 3). OE, overexpression. (**H**) RT-qPCR analysis of indicated mRNAs in MDA-MB-231 cells overexpressing EV or RCOR2 treated with DMSO or 0.2 μM TSA for 24 hours and in combination with 0.1 ng/mL IFN-γ for another 24 hours (*n* = 3). (**I** and **J**) Genome browser snapshots of HA, HDAC1, and LSD1 binding peaks, highlighted in gold-yellow, on *RNF43* (**I**) and *CIITA* (**J**) in control, RCOR2-OE, and RCOR2-KO MDA-MB-231 cells (*n* = 2). (**K** and **L**) ChIP-qPCR assay showing relative H3K4me2 (**K**) and H4K16Ac (**L**) occupancy on *RNF43* and *CIITA* in parental and RCOR2-KO MDA-MB-231 cells. Data represent mean ± SEM. *P* values were determined by 1-way ANOVA with Tukey’s test (**G** and **H**) or Dunnett’s test (**C** and **F**), 2-way ANOVA with Tukey’s test (**K** and **L**), and 2-tailed Student’s *t* test (**A** and **B**). *P < 0.05; **P < 0.01; ***P < 0.001; ****P < 0.0001.

Two types of histone modifiers, LSD1 and HDAC1/2, are associated with RCOR2 in the complex (36). Treatment with an LSD1 inhibitor, GSK-LSD1 (50 μM), significantly induced the expression of *RNF43*, but not *CIITA* and MHC-II heavy chain genes, whereas an HDAC inhibitor, Trichostatin A (TSA) (0.2 μM), had an opposite effect on the expression of these genes in MDA-MB-231 cells (Figure 5, A and B). These results were confirmed by genetic KO of LSD1, HDAC1, or HDAC2 in MDA-MB-231 cells treated with or without 0.1 ng/mL IFN-γ (Figure 5, C–F). We further found that GSK-LSD1 treatment blocked RCOR2-induced *RNF43* repression in MDA-MB-231 cells (Figure 5G), whereas TSA treatment caused *CIITA* derepression in MDA-MB-231 cells overexpressing RCOR2 (Figure 5H). These findings indicate that RCOR2 suppresses *RNF43* and *CIITA* through LSD1 and HDAC1/2, respectively.

To support epigenetic regulation of RNF43 and CIITA by the RCOR2 complex, we next performed ChIP sequencing (ChIP-Seq) in MDA-MB-231 cells overexpressing HA-RCOR2 and detected 2 strong RCOR2 binding peaks at the second intron of the *RNF43* gene and 3 strong RCOR2 binding peaks at the promoter of the *CIITA* gene (Figure 5, I and J). RCOR2 occupancies were detected at the genome nearest to *HLA-DMA* and *HLA-DMB*, but not other MHC-II heavy chain genes (Supplemental Figure 5, A–C), further supporting indirect repression of MHC-II by RCOR2. Consistently, RCOR2 KO1 selectively increased H3K4me2 enrichment on *RNF43* intron 2 and H4K16ac enrichment on the *CIITA* promoter in MDA-MB-231 cells (Figure 5, K and L). Both LSD1 and HDAC1 were colocalized with RCOR2 at *RNF43* and *CIITA*, but their enrichment was not affected by RCOR2 KO1 in MDA-MB-231 cells (Figure 5, I and J), suggesting that RCOR2 is not involved in recruitment of LSD1 and HDAC1 to *RNF43* and *CIITA* genes and that the enzymatic activity of LSD1 and HDAC1/2 is selectively stimulated on *RNF43* and *CIITA*. Together, these findings indicate that RCOR2 reduces H3K4me2 and H4K16ac to suppress the expression of *RNF43* and *CIITA*, respectively.

### Tumoral CIITA/MHC-II silencing is responsible for RCOR2-induced tumor immune evasion.

**Figure 6:**
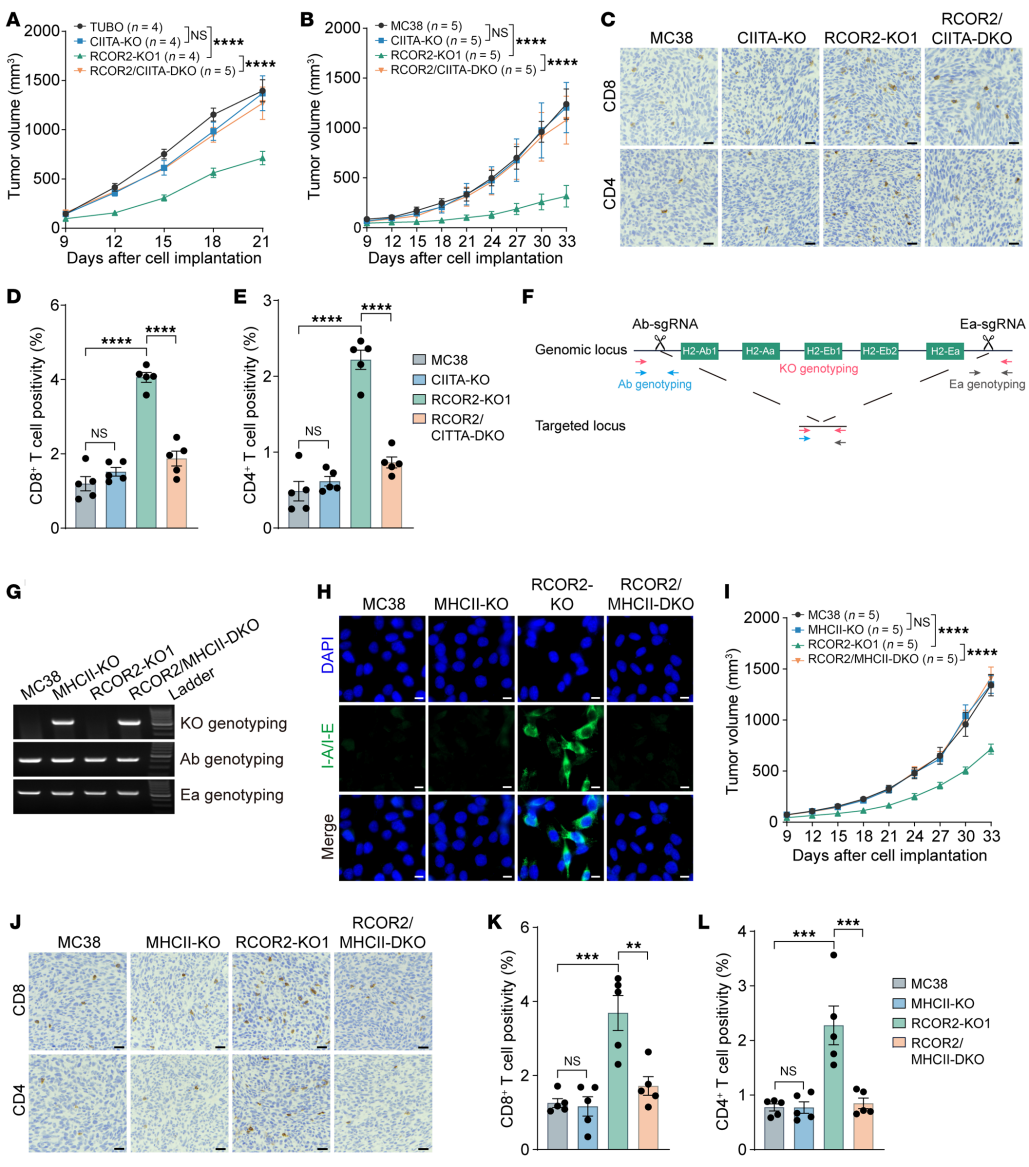
RCOR2 promotes tumor immune evasion by suppressing CIITA and MHC-II. (**A** and **B**) Growth of parental, RCOR2-KO1, CIITA-KO, and RCOR2/CIITA–DKO TUBO (**A**) or MC38 (**B**) tumors in BALB/c or C57BL/6J mice. (**C**–**E**) CD4 and CD8 IHC in parental, RCOR2-KO1, CIITA-KO, and RCOR2/CIITA–DKO MC38 tumors (**C**); the percentage of T cells is quantified (**D** and **E**; *n* = 5). Scale bars: 25 μm. (**F**) Scheme of MHC-II KO using CRISPR/Cas9. (**G**) Genotyping of MHC-II KO in parental, RCOR2-KO1, MHCII-KO, and RCOR2/MHCII–DKO MC38 cells. (**H**) Representative immunostaining of I-A/I-E in parental, RCOR2-KO1, MHCII-KO, and RCOR2/MHCII–DKO MC38 cells treated with 5 ng/mL IFN-γ for 24 hours. Scale bars: 10 μm. (**I**) Growth of parental, RCOR2-KO1, MHCII-KO, and RCOR2/MHCII–DKO MC38 tumors in C57BL/6J mice. (**J**–**L**) CD4 and CD8 IHC in parental, RCOR2-KO1, and RCOR2/MHCII–DKO MC38 tumors (**J**); the percentage of T cells is quantified (**K** and **L**; *n* = 5). Scale bars: 25 μm. Data represent mean ± SEM. *P* values were determined by 1-way ANOVA with Tukey’s test (**D**, **E**, **K**, and **L**) and 2-way ANOVA with Tukey’s test (**A**, **B**, and **I**). **P < 0.01; ***P < 0.001; ****P < 0.0001.

Next, we studied whether CIITA silencing regulates RCOR2-induced tumor immune evasion. Parental, RCOR2-KO1, CIITA-KO, and RCOR2/CIITA–double-KO (DKO) TUBO cells were orthotopically implanted into the mammary fat pad of female BALB/c mice. CIITA KO reversed tumor reduction conferred by RCOR2 loss in mice, even though CIITA KO alone had no effect on tumor growth (Figure 6A). Similar results were observed in the MC38 tumor mouse model (Figure 6B). Increased infiltration of CD4^+^ and CD8^+^ T cells was also reversed in tumors when CIITA was co-deleted with RCOR2 (Figure 6, C–E). These results indicate that CIITA silencing is responsible for RCOR2-induced T cell evasion and tumor growth in syngeneic mouse models.

To further determine whether loss of MHC-II–mediated antigen presentation controls RCOR2-mediated immune escape, we deleted all five of the classic mouse MHC-II heavy chain genes in parental and RCOR2-KO1 MC38 tumor cells using the CRISPR/Cas9 technique (Figure 6F). A genotyping test showed that all five MHC-II heavy chain genes were deleted from one allele in both parental and RCOR2-KO1 MC38 cells (Figure 6G), which was sufficient to deplete their proteins (Figure 6H). MHC-II protein depletion completely abolished tumor reduction caused by RCOR2 loss in mice (Figure 6I), which phenocopied CIITA loss (Figure 6, A and B). Increased infiltration of CD4^+^ and CD8^+^ T cells was also reversed in RCOR2/MHC-II–DKO tumors (Figure 6, J–L). These results indicate that MHC-II silencing is responsible for RCOR2-induced T cell evasion and tumor growth in mice.

**Figure 7:**
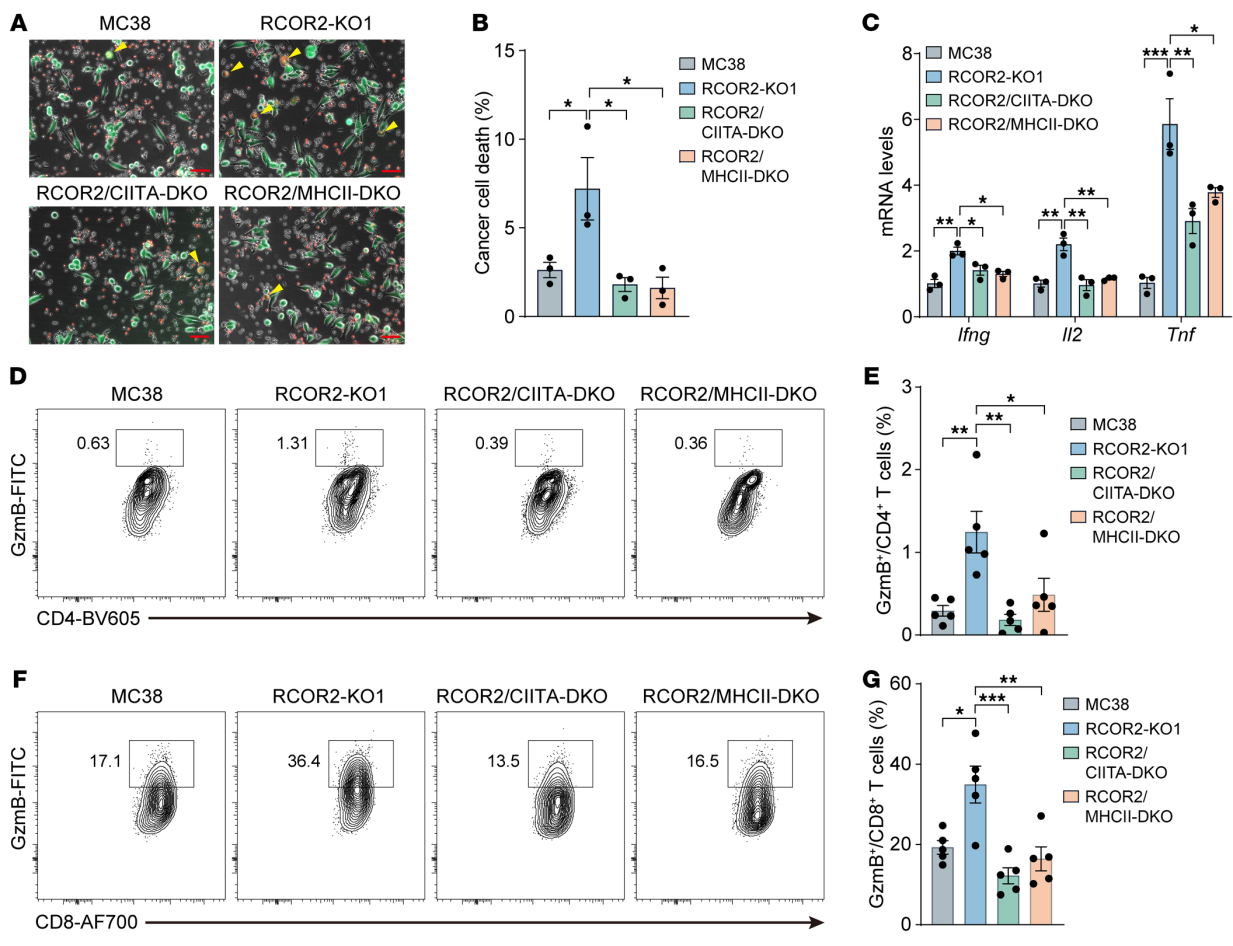
Tumoral RCOR2 impedes activation of intratumoral CD4^+^ and CD8^+^ T cells through CIITA/MHC-II silencing. (**A** and **B**) CD4^+^ T cell killing assay in coculture with parental, RCOR2-KO1, RCOR2/CIITA–DKO, and RCOR2/MHCII–DKO MC38 cells (*n* = 3). Representative images are shown in **A**. Cancer cell death is quantified in **B**. Scale bars: 50 μm. (**C**) RT-qPCR analysis of indicated mRNAs in CD4^+^ T cells after coculture with parental, RCOR2-KO1, RCOR2/CIITA–DKO, and RCOR2/MHCII–DKO MC38 cells (*n* = 3). (**D** and **E**) Flow cytometry analysis of GzmB-expressing CD4^+^ T cells in parental, RCOR2-KO1, RCOR2/CIITA–DKO, and RCOR2/MHCII–DKO MC38 tumors (*n* = 5). Representative gating is shown in **D**. The percentage of GzmB-expressing CD4^+^ T cells is quantified in **E**. (**F** and **G**) Flow cytometry analysis of GzmB-expressing CD8^+^ T cells in parental, RCOR2-KO1, RCOR2/CIITA–DKO, and RCOR2/MHCII–DKO MC38 tumors (*n* = 5). Representative gating is shown in **F**. The percentage of GzmB-expressing CD8^+^ T cells is quantified in **G**. Data represent mean ± SEM. *P* values were determined by 1-way ANOVA with Tukey’s test (**B**, **C**, **E**, and **G**). *P < 0.05; **P < 0.01; ***P < 0.001.

To determine whether RCOR2 impairs cytotoxicity of CD4^+^ T cells through CIITA/MHC-II silencing, we performed CD4^+^ T cell killing assay by coculturing CD4^+^ T cells isolated from OT-II mouse spleen with parental or RCOR2-KO1 MC38 cells pretreated with the OVA323-39 peptide at the ratio of 10:1. The number of dead RCOR2-KO1 MC38 cells, which are shown in yellow, was significantly increased after coculture with CD4^+^ T cells as compared with parental MC38 cells, which was prevented by loss of CIITA or MHC-II (Figure 7, A and B). Under conditions of coculture with RCOR2-KO1 MC38 cells, CD4^+^ T cells expressed higher mRNA levels of cytotoxic cytokines, including IFN-γ and TNF-α and the T cell fate activator IL-2, than those in the other 3 coculture groups (Figure 7C). Consistently, we showed that loss of tumoral RCOR2 significantly increased granzyme B–expressing (GzmB-expressing) CD4^+^ and CD8^+^ T cells in MC38 tumors, which was reversed by co-deletion of CIITA or MHC-II (Figure 7, D–G), suggesting that tumoral RCOR2 impedes activation of cytotoxic CD4^+^ and CD8^+^ T cells in tumors through CIITA/MHC-II silencing. Collectively, these findings indicate that RCOR2 downregulates MHC-II–mediated antigen presentation in cancer cells, leading to tumor escape from T cell immunosurveillance.

### Activation of Wnt/β-catenin signaling is responsible for RCOR2-induced tumor cell plasticity.

**Figure 8:**
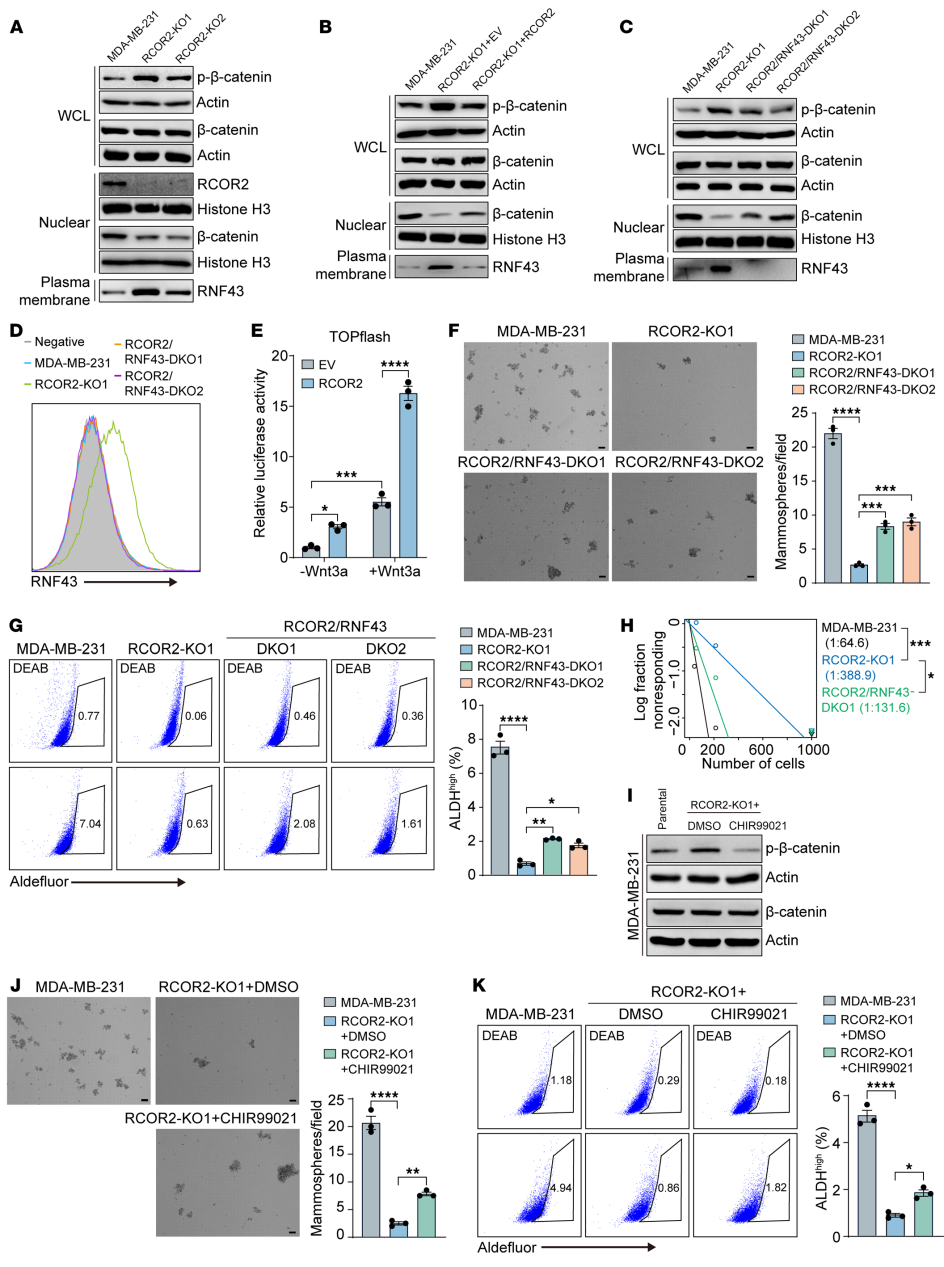
RCOR2 enhances BCSC stemness by attenuating RNF43-mediated Wnt/β-catenin inactivation. (**A**) Immunoblot of indicated proteins in parental and RCOR2-KO1 or -KO2 MDA-MB-231 cells. (**B**) Immunoblot of indicated proteins in parental, RCOR2-KO1, and RCOR2-rescue MDA-MB-231 cells. (**C**) Immunoblot of indicated proteins in parental, RCOR2-KO1, and RCOR2/RNF43–DKO MDA-MB-231 cells. (**D**) Flow cytometry analysis of RNF43 protein in parental, RCOR2-KO1, and RCOR2/RNF43–DKO MDA-MB-231 cells. (**E**) TOPFlash assay in HEK293T cells transfected with EV or RCOR2 and treated with Wnt3a for 48 hours (*n* = 3). (**F**) Mammosphere formation assay of parental, RCOR2-KO1, and RCOR2/RNF43–DKO MDA-MB-231 cells. Representative mammosphere images are shown (left), and mammosphere numbers are quantified (right; *n* = 3). (**G**) Flow cytometry analysis (left) and quantification (right) of ALDH^hi^ cells in parental, RCOR2-KO1, and RCOR2/RNF43–DKO MDA-MB-231 cells (*n* = 3). (**H**) Limiting dilution assay of parental, RCOR2-KO1, and RCOR2/RNF43–DKO1 MDA-MB-231 cells in NSG mice. (**I**) Immunoblot of indicated proteins in parental and RCOR2-KO1 MDA-MB-231 cells treated with DMSO or 1 μM CHIR99021 for 48 hours. (**J**) Mammosphere formation assay of parental and RCOR2-KO1 MDA-MB-231 cells treated with DMSO or 1 μM CHIR99021. Representative mammosphere images are shown (left), and mammosphere numbers are quantified (right; *n* = 3). (**K**) Flow cytometry analysis (left) and quantification (right) of ALDH^hi^ cells in parental and RCOR2-KO1 MDA-MB-231 cells treated with DMSO or 1 μM CHIR99021 for 48 hours (*n* = 3). Data represent mean ± SEM. *P* values were determined by 1-way ANOVA with Tukey’s test (**F**, **G**, **J**, and **K**), 2-way ANOVA with Tukey’s test (**E**), and χ^2^ test (**H**). *P < 0.05; **P < 0.01; ***P < 0.001; ****P < 0.0001. Scale bars: 100 μm.

Next, we studied whether RCOR2 controls activation of Wnt/β-catenin signaling in cancer cells. Along with elevated membrane-bound RNF43, loss of RCOR2 increased β-catenin phosphorylation but decreased nuclear β-catenin levels in MDA-MB-231 cells, both of which were reversed by either RCOR2 re-expression (Figure 8, A and B) or RNF43 deletion (Figure 8, C and D). Consistently, forced expression of RCOR2 significantly increased the basal β-catenin luciferase reporter activity in transfected HEK293T cells, which was further enhanced after cells were treated with Wnt3a protein for 24 hours (Figure 8E). These findings indicate that RCOR2 enhances activation of Wnt/β-catenin signaling through RNF43 silencing.

We next examined whether RNF43 silencing contributes to RCOR2-induced breast tumor plasticity. RNF43 KO1 or KO2 counteracted RCOR2 loss to partially restore MDA-MB-231 mammospheres and ALDH^hi^ BCSCs (Figure 8, F and G). Reduced tumor initiation frequency by RCOR2 loss in mice was also rescued when RNF43 was co-deleted (Figure 8H). To further confirm that activation of Wnt/β-catenin signaling is responsible for RCOR2-induced breast cancer cell plasticity, we treated RCOR2-KO1 MDA-MB-231 cells with a specific GSK3 inhibitor, CHIR99021 (1 μM), which can bypass RNF43 to activate β-catenin. As expected, CHIR99021 treatment blocked increased phosphorylation of β-catenin in RCOR2-KO1 MDA-MB-231 cells (Figure 8I). Activation of β-catenin by CHIR99021 partially rescued mammospheres and ALDH^hi^ BCSCs (Figure 8, J and K). Together, these findings indicate that RCOR2 enhances Wnt/β-catenin activation by RNF43 silencing, leading to increased breast cancer plasticity.

### Targeting RCOR2 potentiates anti–PD-1 blockade therapy in mice.

**Figure 9:**
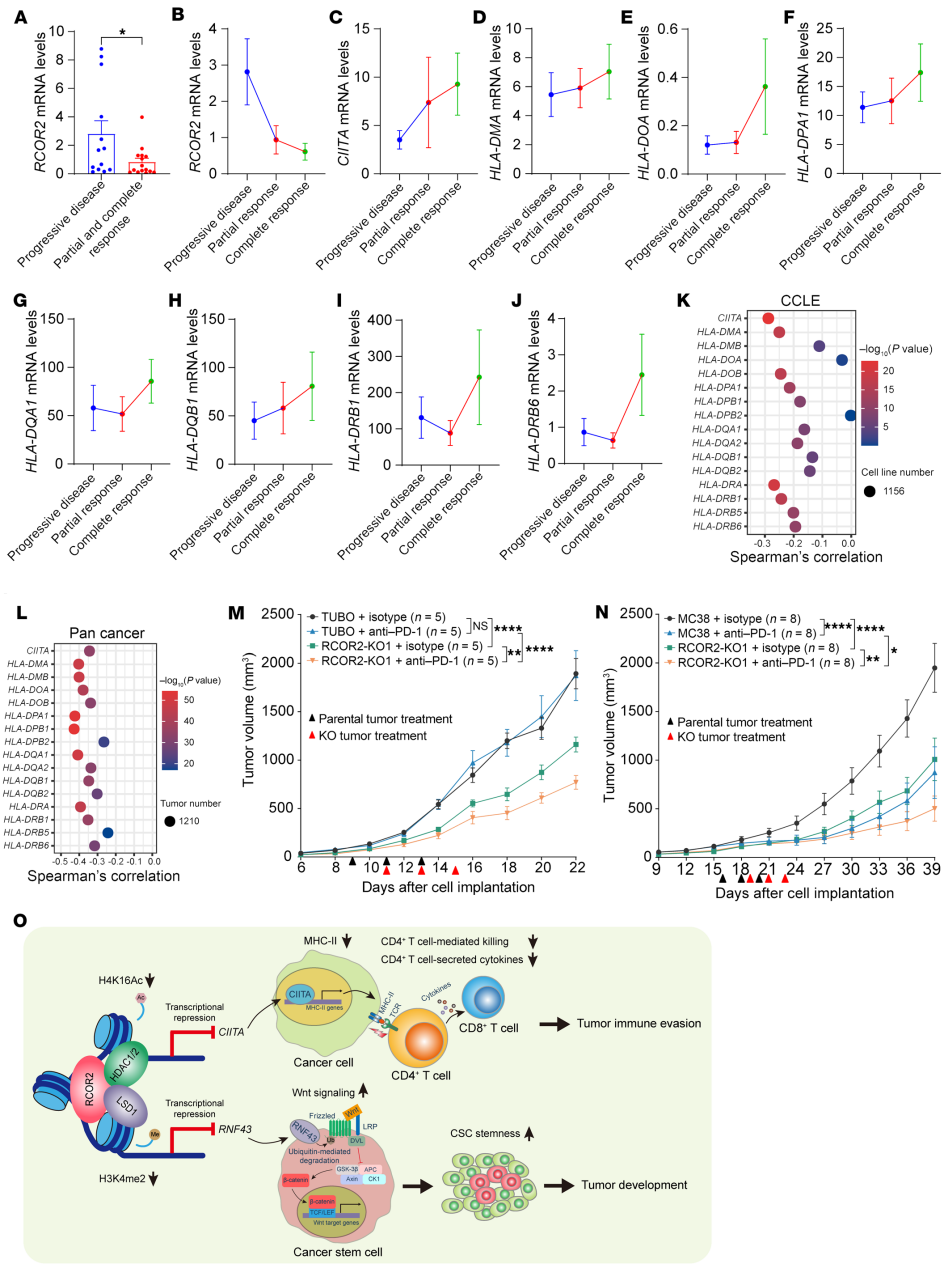
RCOR2 is inversely correlated with response to anti–PD-1 therapy in patients, and its loss potentiates anti–PD-1 treatment in mice. (**A**–**J**) Analysis of indicated mRNAs in melanoma from complete responders (*n* = 5), partial responders (*n* = 10), and non-responders (*n* = 13) to anti–PD-1 therapy. RNA-Seq data were retrieved from GSE78220. (**K**) Spearman’s correlation analysis between *RCOR2* and *CIITA*/MHC-II heavy chain gene mRNAs in 1,156 human cancer cell lines. Data were retrieved from the Cancer Cell Line Encyclopedia (CCLE). (**L**) Spearman’s correlation analysis between *RCOR2* and *CIITA*/MHC-II heavy chain gene mRNAs in 1,210 human tumors. Data were retrieved from the International Cancer Genome Consortium/TCGA Pan-Cancer Analysis of Whole Genomes Consortium at cBioPortal. (**M** and **N**) Growth of parental and RCOR2-KO1 TUBO (**M**) and MC38 (**N**) tumors in BALB/c and C57BL/6J mice, respectively, to which control IgG or anti–PD-1 antibody was administered. (**O**) A proposed mechanistic model of RCOR2-dependent tumor cell plasticity and immune evasion. Data represent mean ± SEM. *P* values were determined by 2-way ANOVA with Tukey’s test (**M** and **N**) and 2-tailed Student’s *t* test (**A**). *P < 0.05; **P < 0.01; ****P < 0.0001.

The transcriptomic analysis revealed a significant decrease in *RCOR2* mRNA expression in melanoma from patients who achieved a partial or complete response to anti–PD-1 ICB, as compared with those who did not respond to the treatment (Figure 9A). The highest levels of *RCOR2* mRNA were detected in 13 non-responding melanoma tumors, whereas the lowest levels of *RCOR2* mRNA were found in 5 completely responding melanoma tumors (Figure 9B). In contrast, the mRNA expression of *CIITA* and MHC-II heavy chain genes was gradually increased from non-responding to partially responding to completely responding melanoma tumors (Figure 9, C–J). Consistently, *RCOR2* inversely correlated with *CIITA* and most MHC-II heavy chain genes in 1,156 cancer cell lines and 1,210 pan-cancers (Figure 9, K and L). These results confirm negative regulation of CIITA and MHC-II by RCOR2 in human cancers and suggest negative correlation between RCOR2 levels and responses to anti–PD-1 blockade therapy.

To assess whether targeting tumoral RCOR2 can improve anti–PD-1 ICB, we orthotopically implanted parental or RCOR2-KO1 TUBO cells into the mammary fat pad of female BALB/c mice, and anti–PD-1 antibody or control antibody was intraperitoneally administered to mice when the volume of parental and RCOR2-KO1 tumors reached about 100 mm^3^. Treatment with anti–PD-1 antibody had no therapeutic response in parental TUBO tumors but significantly inhibited RCOR2-KO1 tumor growth in mice (Figure 9M). An enhanced tumor-inhibitory effect of anti–PD-1 antibody and RCOR2 KO1 combination was also achieved in the MC38 mouse model (Figure 9N). Collectively, these results indicate that RCOR2 is a valuable therapeutic target and biomarker that can predict a response to anti–PD-1 ICB in cancers.

## Discussion

In this study, we uncover a dual role of the RCOR2 complex in tumor cell plasticity and immunogenicity leading to tumor initiation and progression in mice. The underlying mechanism involves two distinct epigenetic signaling pathways controlled by RCOR2 and its associated histone modifiers, LSD1 and HDAC1/2 (Figure 9O). The RCOR2/LSD1 sub-axis suppresses *RNF43* transcription to activate Wnt/β-catenin signaling in tumor cells, whereas the RCOR2/HDAC1/2 sub-axis inhibits CIITA and MHC-II expression in tumor cells to block activation of CD4^+^ and CD8^+^ T cells (Figure 9O). As such, RCOR2 is a central regulator that integrates cancer cell–intrinsic plasticity signals and extrinsic immune surveillance signals in tumors. Notably, loss of RCOR2 robustly improves anti–PD-1 blockade therapy in mouse models of cancer. Collectively, our work identifies a “two birds with one stone” effect for RCOR2 in cancers and establishes a valuable framework to simultaneously target tumor cell plasticity and immunogenicity for the better treatment of human cancers.

Our present studies show that RCOR2 increases breast cancer stemness, suggesting a conserved function of RCOR2 from normal stem cells to cancer stem cells. The rescue effect of RCOR2 on mammosphere formation is modest, possibly because of its weak ability to reprogram differentiated non-BCSCs into BCSCs. The expression levels of RCOR2 are higher in BCSCs compared with non-BCSCs. Cancer stem cells frequently reside at the hypoxic area within tumors (37), where the transcription factor hypoxia-inducible factor (HIF) is activated (38). RCOR2 is known to be induced by HIF-1 (39), suggesting that HIF-1 may be involved in RCOR2 upregulation in BCSCs. Interestingly, breast tumor cell plasticity induced by RCOR2 is LSD1 dependent. RNF43 silencing by the RCOR2-LSD1 axis is responsible for maintenance of breast cancer stemness. RNF43 is frequently mutated in ovarian, colon, and pancreatic cancers and functions as a tumor suppressor (40–42). Our studies suggest that RNF43 silencing by RCOR2 is an additional mechanism to diminish its tumor suppressor function in wild-type tumors. We show a partial rescue effect of RNF43 silencing or CHIR99021 treatment on stemness of RCOR2-null breast cancer cells, although β-catenin activity is fully restored by these two interventions. These results suggest that, in addition to RNF43–Wnt/β-catenin signaling, other mechanisms are also involved in RCOR2-induced tumor plasticity, which require further investigation. Nevertheless, we identify activation of Wnt/β-catenin signaling as the mechanism of RCOR2-induced breast cancer stemness and tumor initiation, which offers mechanistic insights into RCOR2-dependent stem cell biology.

MHC-II molecules are underexpressed in the majority of human tumors (27). CIITA is a master regulator of MHC-II (17) and is regulated by multiple factors, including FBXO11, PML, PRMT5, and NFAT5, in mammalian cells (43–46). Given its selective expression pattern in tumor cells, RCOR2 is a specific corepressor of CIITA and MHC-II in tumor cells. Thus, targeting RCOR2 is a valuable therapeutic strategy that can achieve selective tumor cell death with less immunotoxicity to normal tissues. We further show that HDAC1 and HDAC2, but not LSD1, are responsible for RCOR2-dependent transcriptional suppression of *CIITA* in cancer cells. Interestingly, both LSD1 and HDAC1 bind to *RNF43* and *CIITA*, and their chromatin occupancy is not controlled by RCOR2, suggesting that an additional factor determines the specificity of RCOR2-induced *RNF43* and *CIITA* silencing by selectively stimulating LSD1 and HDAC1/2 activities.

While CD4^+^ T cells are traditionally considered as helper cells for activation of CD8^+^ T cells (11), emerging studies from the past decade show that a subpopulation of CD4^+^ T cells exhibits cytotoxicity against tumors with high levels of MHC-II (12–16). MHC-II–abundant APCs, including dendritic cells, macrophages, and B cells, play a central role in CD4^+^ T cell activation in the tumor microenvironment (18, 19). Our studies show that CD4^+^ T cells also have a direct cytolytic role against tumor cells, which is non-cell-autonomously activated by the RCOR2–HDAC1/2–CIITA–MHC-II axis in tumor cells. These results suggest that loss of RCOR2 can enhance the transformation of tumor cells into APCs to activate CD4^+^ T cells, although the specific tumor antigens involved in this context remain unidentified. Previous studies showed that Th1 CD4^+^ T cells exhibit cytotoxic activity and produce cytotoxic cytokines (12–14). Similarly, we detect increased IFN-γ, TNF-α, and IL-2 expression in CD4^+^ T cells after coculture with RCOR2-KO tumor cells. Loss of tumoral RCOR2 increases GzmB-expressing CD4^+^ T cells in tumors; however, the precise subtype of CD4^+^ T cells activated by loss of tumoral RCOR2 remains to be investigated. Nevertheless, our findings highlight that a RCOR2-based therapeutic approach can enhance CD4^+^ T cell activity, thereby boosting antitumor immunity.

MHC-II also stimulates CD4^+^ regulatory T cells, which function as immunosuppressive factors contributing to immune evasion in tumors (47). We show that loss of tumoral RCOR2 has no effect on enrichment of regulatory T cells in mouse tumor models, excluding a role of CD4^+^ regulatory T cells in RCOR2-mediated immune evasion. Additionally, CD8^+^ T cells are involved in RCOR2-mediated immune evasion. However, RCOR2 fails to regulate MHC-I expression. Thus, our results suggest that, in additional to its role in cytotoxicity of CD4^+^ T cells, loss of tumoral RCOR2 can enhance CD4^+^ T cell helper function, leading to activation of CD8^+^ T cells. Clinical studies have revealed positive correlation of tumoral MHC-II with better survival of cancer patients (23–26). Consistently, we show that RCOR2 is negatively associated with MHC-II molecules in tumors and survival of breast cancer patients. Together, these clinical studies in cancer patients strongly support RCOR2’s role in evading CD4^+^CD8^+^ T cell surveillance. While our studies identify a pivotal role of RCOR2 in CD4^+^CD8^+^ T cell–mediated immune evasion, RCOR2 loss also increases infiltration of B cells in MC38 tumors, another type of lymphocytes involved in antitumor immunity (48). Future investigation regarding B cells will provide advanced insights into RCOR2-mediated immune evasion in tumors.

ICB therapy has achieved tremendous success in cancer treatment; however, numerous cancer patients do not have a durable response to ICB treatment (49, 50). We show that *RCOR2* is negatively associated with anti–PD-1 therapy in melanoma patients. Loss of RCOR2 significantly enhances anti–PD-1 therapeutic efficacy in both immune-hot and -cold tumor models. These findings identify RCOR2 as a key regulator and biomarker of immune evasion and resistance to ICB therapy. Emerging studies have identified many resistance mechanisms of ICB therapy (51). Epithelial-mesenchymal transition (EMT) is one of the key factors that confer resistance to ICB therapies (31, 52). Targeting of both EMT and PD-1 with TGF-β receptor inhibitor and anti–PD-1 antibody has been developed; however, this rational combination therapy shows limited success in improving clinical outcomes for lung cancer patients (53). Specific therapeutic targets and biomarkers may help identify patients who could benefit from targeting of both tumor plasticity and immune evasion. Collectively, our studies suggest that targeting RCOR2 can inhibit not only tumor plasticity but also immune evasion, potentially eradicating malignant diseases and substantially advancing cancer treatment.

### Limitations of the study.

In this study, we use a genetic approach to establish proof-of-concept that targeting RCOR2 can achieve a “two birds with one stone” effect for the better treatment of cancers. This genetic approach may encounter challenges when applied to clinical studies. The future development of a specific small-molecule inhibitor of RCOR2 has the potential to revolutionize cancer treatment regimens.

## Methods

### Sex as a biological variable.

Our study examined male and female animals, and similar findings are reported for both sexes.

### Plasmid constructs.

sgRNAs targeting human *RCOR2*, *LSD1*, *HDAC1*, *HDAC2*, and *RNF43* and mouse *Rcor2*, *Ciita*, and MHC-II heavy chain gene locus (Supplemental Table 1) were designed by the online CRISPR design program CRISPick (54). DNA oligonucleotides of sgRNAs were annealed and cloned into BsmBI-linearized lentiCRISPRv2 vector (Addgene, 52961). Human *RCOR2* cDNA was PCR amplified and cloned into p3×FLAG-CMV-7 (MilliporeSigma), lentiviral cFugw-3×FLAG, or pLL-UBC-2×HA vector. Human *RCOR2* (392–447 aa) cDNA was PCR amplified and cloned into pGex-6P-1 (GE Healthcare) vector.

### Cell culture and lentivirus production.

MDA-MB-231 (a gift from R. Brekken, UT Southwestern, Dallas, Texas, USA), HEK293T (a gift from G.L. Semenza, Johns Hopkins School of Medicine, Baltimore, Maryland, USA), HEK293FT (Thermo Fisher Scientific), MCF-7 (American Type Culture Collection), and TUBO and MC38 (gifts from Yang-Xin Fu, UT Southwestern) cells were cultured in high-glucose DMEM (Sigma-Aldrich) supplemented with heat-inactivated 10% fetal bovine serum (FBS, Sigma-Aldrich) at 37°C in a 5% CO_2_/95% air incubator. Lentivirus was generated in HEK293FT cells as described previously (55).

### Generation of KO, overexpressing, and rescue cell lines.

CRISPR/Cas9–mediated KO in MDA-MB-231, MCF-7, and TUBO cells has been described previously (55). MC38 cells were transfected with sgRNA vector and pcDNA3.1 plasmid (Thermo Fisher Scientific) using Lipofectamine 3000 (Thermo Fisher Scientific). Forty-eight hours after transfection, cells were treated with G418 (500 μg/mL) for 3 days. Single KO cells were selected, amplified, and verified by immunoblot assay and/or PCR genotyping. Multiple KO clones were mixed for further studies. RCOR2-overexpression (OE) or rescue cells were generated by infection of parental or RCOR2-KO cells with lentivirus carrying *RCOR2* cDNA.

### Cell proliferation and colony formation assays.

For cell proliferation assay, MDA-MB-231 cells (2 × 10^5^ cells per well) were seeded onto a 6-well plate and cultured for 24, 48, and 72 hours. The cell number at each time point was determined by trypan blue assay. For colony formation assay, 100 cells were seeded on a 6-well plate and cultured for 12 days. Colonies were washed with PBS, fixed with methanol, and stained with 0.5% crystal violet (MilliporeSigma). After staining, the colonies were gently washed and counted.

### RCOR2 antibody generation and purification.

Glutathione S-transferase (GST)-tagged RCOR2 (392–447 aa) was expressed in *E*. *coli* BL21-Gold (DE3) and purified with glutathione-Sepharose beads (GE Healthcare) as described previously (55). Two milligrams of purified protein was injected into a rabbit for polyclonal RCOR2 antibody generation (YenZym Antibodies). Antisera were collected for RCOR2 antibody purification. Purified GST and GST-RCOR2 (392–447 aa) proteins were bound to glutathione-Sepharose beads (GE Healthcare) and cross-linked by incubation for 30 minutes with 8 mg/mL dimethyl pimelimidate at room temperature. Antisera were consecutively incubated for 1 hour each with cross-linked GST and GST-RCOR2 (392–447 aa) at 4°C, washed, and eluted with 0.1 M glycine (pH 2.5) at room temperature. The eluted antibody was adjusted to pH 7.0 with Tris-HCl (pH 8.0), concentrated using a 10 kDa Amicon Ultra Centrifugal Filter (Millipore), and validated by immunoblot assay in parental and RCOR2-KO cancer cells.

### Immunoblot assay.

Homogenized tissues or cells were lysed in NETN lysis buffer (150 mM NaCl, 1 mM EDTA, 10 mM Tris-HCl [pH 8.0], 0.5% NP-40, and protease inhibitor cocktail) for 30 minutes on ice, followed by sonication. For preparing nuclear and plasma membrane lysate, cells were lysed with FA lysis buffer (150 mM NaCl, 10 mM EDTA, 10 mM Tris-HCl [pH 8.0], 0.25% Triton X-100, and protease inhibitor cocktail). After centrifugation at 850 *g* for 10 minutes at 4°C, supernatants were transferred to fresh tubes. The pellets were collected as nuclear fractions. Then supernatants were centrifuged at 16,000 *g* for 10 minutes at 4°C to collect pellets as plasma membrane fractions. Nuclear and plasma membrane fractions were washed with FA lysis buffer, lysed in NETN lysis buffer, and sonicated. After centrifugation at 16,000 *g* for 10 minutes at 4°C, the supernatant was boiled in 1× Laemmli buffer, and fractionated by SDS-PAGE, followed by immunoblot assay with antibodies listed in Supplemental Table 2.

### IHC assay.

IHC assay was performed by the Dako Autostainer Link 48 system. Briefly, the slides were baked, deparaffinized, and hydrated, followed by antigen retrieval in a Dako PT Link. The tissues were incubated with a peroxidase block, followed by staining with primary antibody: RCOR2 (1:50; homemade), CD8α (1:400; Cell Signaling Technology, catalog 98941), or CD4 (1:100; Cell Signaling Technology, catalog 25229). The staining was visualized using the EnVision FLEX visualization system (Dako). The H-scores of protein staining were calculated using QuPath software (Version 0.2.3, University of Edinburgh.

### Immunostaining assay.

Parental, RCOR2-KO, CIITA-KO, MHC-II–KO, RCOR2/CIITA–DKO, or RCOR2/MHC-II–DKO MC38 cells were seeded onto glass coverslips placed in a 12-well plate and cultured for 48 hours. After washing with PBS, cells were fixed for 20 minutes with 4% paraformaldehyde at room temperature, permeabilized, and blocked for 60 minutes with PBS supplemented with 5% BSA and 0.1% Triton X-100. Cells were then incubated overnight with anti–I-A/I-E antibody (1:500; Thermo Fisher Scientific, catalog 14-5321-82) at 4°C. After washing 3 times with PBST (PBS with 0.1% Tween-20), cells were incubated for 60 minutes with Alexa Fluor 488 goat anti-rat IgG and DAPI in the dark. After washing again 3 times with PBST, cells were mounted with antifade mounting medium. Mounted slides were observed with a Zeiss Axio Observer Z1 fluorescence microscope.

### Sphere formation assay.

MDA-MB-231 or MCF-7 cells were trypsinized to single-cell suspensions, washed with HBSS, resuspended in MammoCult medium (STEMCELL Technologies) with or without DMSO (Sigma-Aldrich) and 1 μM CHIR99021 (SelleckChem), and cultured for 4–7 days on a 6-well ultra-low-attachment plate at 37°C in a 5% CO_2_/95% air incubator. Mammospheres were imaged under a Zeiss Axio Observer Z1 microscope.

PyMT tumorspheres were generated as described previously (56). Briefly, tumors were harvested, cross-cut, washed, and digested for 45 minutes with gentle collagenase/hyaluronidase (STEMCELL Technologies) in a 37°C shaker. After filtering with a 40 μm cell strainer (Thermo Fisher Scientific) and centrifugation at 400 *g* for 5 minutes, single cells were resuspended in DMEM/Ham’s F-12 medium with B-27 supplement (Thermo Fisher Scientific), EGF (20 ng/mL; Sigma-Aldrich), basic fibroblast growth factor (20 ng/mL; STEMCELL Technologies), heparin (4 μg/mL; STEMCELL Technologies), and 1% penicillin/streptomycin/neomycin (Sigma-Aldrich) and plated overnight on a collagen I–coated plate (Thermo Fisher Scientific) at 37°C in a 5% CO_2_/95% air incubator. The next day, cells were trypsinized and reseeded on an ultra-low-attachment dish (Corning) at 37°C in a 5% CO_2_/95% air incubator for continuous incubation for 7 days. Tumorspheres were imaged under a Zeiss Axio Observer Z1 microscope.

### Flow cytometry assay.

MC38 and TUBO tumors were cross-cut into small pieces in PBS, washed, and digested for 45 minutes with collagenase/hyaluronidase/DNase (Sigma-Aldrich) at 37°C. Digested tissues were filtered through a 70 μm cell strainer, treated with red blood cell lysis buffer (Roche), and washed with PBS supplemented with 2% FBS (staining buffer). Cultured cells were trypsinized and dissociated into single-cell suspensions. Single cells were incubated with anti-CD16/32 (anti-FcγIII/II receptor, clone 2.4G2, Bio X Cell) for 10 minutes to block nonspecific binding and then stained with the following antibodies on ice for 30 minutes: anti-CD45, anti-CD3e, anti-CD8, anti-CD4, anti-B220, anti-CD11c, anti-CD11b, anti–Gr-1, anti-F4/80, anti-Ter119, anti-CD31, anti-CD90, anti–I-A/I-E, anti-RNF43, anti-CD44, anti-CD24, or anti-EpCAM antibody (Supplemental Table 2). The fixable viability dye eFluor 506 was used to exclude dead cells. For intracellular staining, cells were fixed with fixation/permeabilization buffer (Invitrogen) on ice for 30 minutes, and then washed twice with 1× permeabilization buffer (Invitrogen). Anti-FOXP3 or anti-GzmB antibody (Supplemental Table 2) was added and incubated for 1 hour on ice. Stained cells were examined on a CytoFLEX Flow Cytometer (Beckman Coulter). Data were analyzed with CytExpert (Beckman Coulter) or FlowJo (Tree Star) software. ALDH^hi^ BCSCs were sorted or quantified in tumors, spheres, and cell cultures as described previously (56).

### Luciferase reporter assay.

HEK293T cells were seeded onto a 48-well plate and transfected with empty vector p3×FLAG-CMV-7 or 3×FLAG-RCOR2, M50 Super 8x TOPFlash reporter plasmid (Addgene, 12456), and control pSV-Renilla reporter plasmid. Twenty-four hours later, cells were treated with or without 100 ng/mL Wnt3a for 48 hours. The firefly and Renilla luciferase activities were measured by the Dual-Luciferase Assay System (Promega).

### RT-qPCR assay.

Total RNA was isolated from cultured cells using TRIzol (Thermo Fisher Scientific), treated with DNase I (Thermo Fisher Scientific), and then subjected to cDNA synthesis with the iScript cDNA Synthesis Kit (Bio-Rad). qPCR was performed with the specific primers (Supplemental Table 3) and iTaq Universal SYBR Green Supermix (Bio-Rad) and normalized to the internal control 18S RNA as described previously (55).

### RNA-Seq assay.

Total RNA was isolated from cultured parental and RCOR2-KO cells using the RNeasy Mini Kit and treated with DNase (QIAGEN). The quality of total RNA was confirmed with an RNA integrity number score of 8.5 or higher by the Agilent TapeStation 4200. RNA-Seq libraries were prepared with KAPA mRNA Hyper Prep (Roche) and sequenced with Illumina NextSeq 2000. Bioinformatics analysis was performed as described previously (55).

### ChIP-Seq and ChIP-qPCR assay.

Parental, RCOR2-KO, HA-EV, and HA-RCOR2 MDA-MB-231 cells were cross-linked with PBS supplemented with 2 mM disuccinimidyl glutarate (Covachem) and 1 mM MgCl_2_ for 45 minutes at room temperature. After washing 3 times with PBS, cells were cross-linked with 1% formaldehyde for 10 minutes at room temperature and quenched in 0.125 M glycine. Cells were lysed in cell lysis buffer (10 mM Tris-HCl [pH 8.0], 10 mM EDTA, 100 mM NaCl, 0.25% Triton X-100, protease inhibitor cocktail). The nuclei were lysed in nuclear lysis buffer (50 mM HEPES-KOH [pH 7.5], 1 mM EDTA, 150 mM NaCl, 1% Triton X-100, 0.1% sodium deoxycholate, 1% SDS, protease inhibitor cocktail), and chromatin was pelleted by centrifugation at 21,000 *g* for 30 minutes at 4°C. The chromatin was then sonicated and subjected to IP overnight in the presence of Protein G Dynabeads (Thermo Fisher Scientific) with antibodies against HA (Cell Signaling Technology, catalog 3724), HDAC1 (Bethyl Laboratories, catalog A300-713A), H3K4me2 (Cell Signaling Technology, catalog 9725), and H4K16ac (Cell Signaling Technology, catalog 13534) or control rabbit IgG (Cell Signaling Technology, catalog 2729) at 4°C. Precipitated chromatin DNA was extensively washed and eluted with the freshly prepared elution buffer (50 mM Tris-HCl [pH 7.5], 10 mM EDTA, 1% SDS). ChIP DNA was subjected to proteinase K treatment at 42°C for 2 hours, reverse-cross-linked at 67°C for 6 hours, treated with RNase A, and purified with phenol/chloroform/isoamyl alcohol (25:24:1, vol/vol). ChIP-Seq libraries were prepared with NEBNext Ultra II DNA Library Prep (New England Biolabs) and sequenced with Illumina NextSeq 2000. Bioinformatics analysis was performed as described previously (55). For qPCR assay, ChIP DNA was quantified by real-time qPCR with the specific primers (Supplemental Table 4). Fold enrichment was calculated based on Ct as 2^–Δ(ΔCt)^, where ΔCt = Ct_IP_ – Ct_input_ and Δ(ΔCt) = ΔCt_antibody_ – ΔCt_IgG_.

### CUT&RUN assay.

5 × 10^5^ parental or RCOR2-KO MDA-MB-231 cells were harvested and washed twice with wash buffer (20 mM HEPES [pH 7.5], 150 mM NaCl, 0.25% 0.5 mM spermidine, protease inhibitor cocktail). Concanavalin A–conjugated beads (Epicypher) were activated in binding buffer (20 mM HEPES [pH 7.5], 10 mM KCl, 1 mM CaCl_2_, 1 mM MnCl_2_) and added into cell suspensions for incubation of 10 minutes at room temperature. Cell-beads slurries were resuspended in antibody buffer (wash buffer supplemented with 0.025% digitonin and 2 mM EDTA) with anti-LSD1 antibody (Cell Signaling Technology, catalog 2184) and incubated overnight at 4°C. After washing twice with Dig-wash buffer (wash buffer with 0.025% digitonin), slurries were incubated with pAG-MNase (Epicypher) for 10 minutes at room temperature, washed twice with Dig-wash buffer, and incubated with 2 mM CaCl_2_ for 2 hours at 4°C. pAG-MNase digestion was terminated by incubation with stop buffer (340 mM NaCl, 20 mM EDTA, 4 mM EGTA, 50 μg/mL RNase A, 50 μg/mL glycogen) for 10 minutes at 37°C. Cleaved chromatin was released, followed by treatment with proteinase K and purification with phenol/chloroform/isoamyl alcohol (25:24:1, vol/vol; Invitrogen). Sequencing libraries were prepared with NEBNext Ultra II DNA Library Prep (New England Biolabs) and sequenced with the Illumina NextSeq 2000. Bioinformatics analysis was performed as described previously (55).

### T cell killing assay.

1.5 × 10^4^ parental, RCOR2-KO, RCOR2/CIITA–DKO, or RCOR2/MHCII–DKO MC38 cells were labeled with CFSE dye (green fluorescence, BioLegend), seeded onto 48-well plates, and cultured for 24 hours in the presence of 5 ng/mL IFN-γ. OVA_323–339_ peptide (1 μg/mL; GenScript) was added into medium 4 hours before T cell coculture. OT-II CD4^+^ T cells were isolated from the spleens of OT-II mice [B6.Cg-Tg(TcraTcrb)425Cbn/J, The Jackson Laboratory] using an EasySep Mouse CD4^+^ T Cell Isolation Kit (STEMCELL Technologies). Purified OT-II CD4^+^ T cells were cocultured with tumor cells at a ratio of 10:1 in RPMI 1640 medium supplemented with 10% FBS, 1× GlutaMAX (Gibco), 1 μg/mL OVA_323–339_ peptide, and Incucyte Cytotox Dye (red fluorescence, Essen Bioscience) for 8 hours. Cells were imaged by Zeiss Axio Observer Z1 microscope. Dead cancer cells were identified by both green and red fluorescence positivity. After imaging, CD4^+^ T cells were collected and subjected to RT-qPCR assay for expression analysis of cytokines.

### Mouse studies.

NSG, C57BL/6J, BALB/c, *Rcor2^fl/fl^* (B6.129-*Rcor2^tm1.1Gman^*/J), MMTV-PyMT [B6.FVB-Tg(MMTV-PyVT)634Mul/LellJ], and CD4-KO (B6.129S2-Cd4^tm1Mak^/J) mice were purchased from The Jackson Laboratory. K14-Cre mice [Tg(KRT14-cre)1Amc/J] were received from the L. Le laboratory (UT Southwestern).

*Rcor2^fl/fl^* mice were crossed with MMTV-PyMT and K14-Cre mice. The primers for mouse genotyping are listed in Supplemental Table 5. Tumor initiation time was determined with palpation and measurement (tumor diameter ≥ 2 mm) in the MMTV-PyMT mouse model. All tumors were harvested, counted, and weighed at postnatal day 155.

For limiting dilution assay, different numbers of cells suspended in 100 μL of PBS/Matrigel (1:1; Corning) were implanted into the second left mammary fat pad of female NSG mice. Tumor onset was determined with palpation and measurement (tumor diameter ≥ 2 mm) 30 days after inoculation. Mice were subcutaneously administered with 17β-estradiol (1 μmol/mouse) daily after MCF-7 cell implantation. ELDA software was used to calculate tumor initiation frequency (57).

MDA-MB-231 (2 × 10^6^), TUBO (1 × 10^5^), and their derivative cell lines in 100 μL PBS/Matrigel (1:1; Corning) were implanted into the second left mammary fat pad of 6- to 8-week-old female NSG or BALB/c mice. MC38 (1 × 10^5^) and its derivative cell lines in 100 μL PBS/Matrigel (1:1; Corning) were implanted subcutaneously into the left flank of male NSG, C57BL/6J, or CD4-KO mice. Tumor volume was measured with a caliper every 3 days from day 6 to day 67 after cell implantation and calculated according to the formula: volume = 0.52 × length × height × width.

For CD8^+^ and CD4^+^ T cell depletion, anti-CD8b mAb (clone 53-5.8, Bio X Cell), anti-CD4 mAb (clone GK1.5, Bio X Cell), or control rat immunoglobulin (Bio X Cell) was injected intraperitoneally at 200 μg/mouse 2 days before cell implantation and 1, 4, and 11 days after cell implantation. For anti–PD-1 antibody treatment, 1 × 10^5^ parental and RCOR2-KO MC38 or TUBO cells were implanted into C57BL/6J or BALB/c mice as above. When the tumor volume reached about 100 mm^3^, 100 μg/mouse anti–PD-1 mAb (clone 29F.1A12, Bio X Cell) or control rat immunoglobulin (Bio X Cell) was administered intraperitoneally every 2 days for a total of 3 times.

### Statistics.

Statistical analysis was performed by 2-tailed Student’s *t* test between 2 groups, and 1- or 2-way ANOVA with multiple testing corrections within multiple groups. Kaplan-Meier survival curves were analyzed by log-rank test. RNA-Seq, ChIP-Seq, and CUT&RUN were repeated twice. The number of biological samples/experiments is shown in figures or figure legends. Data represent mean ± SEM from 3 independent experiments. *P* values less than 0.05 were considered significant.

### Study approval.

Animal experiments were approved by the Animal Care and Use Committee at UT Southwestern Medical Center. The deidentified human tumor tissues were approved by the Institutional Review Board at UT Southwestern Medical Center with informed written consent.

### Data availability.

All data are available in the main text and the supplemental materials. The ChIP-Seq data were deposited at the NCBI’s Gene Expression Omnibus (GEO) with accession number GSE270024. CUT&RUN data were deposited at GEO with accession number GSE269916. The RNA-Seq data were deposited at GEO with accession number GSE270022. Values for all data points in graphs are reported in the Supporting Data Values file. Raw blot data are reported in the full unedited blot and gel images file.

## Author contributions

WL, YW, and LB conceived the study; WL and YW analyzed the data and wrote the paper; LB performed most experiments, analyzed the data, and wrote the paper; MZ generated plasmids; ML performed cell sorting; AK and CX performed bioinformatics analysis; and YP provided tumor tissues. All authors read and approved the manuscript.

## Supplementary Material

Supplemental data

Unedited blot and gel images

Supporting data values

## Version 1 07/03/2025

In-Press Preview

## Version 2 09/16/2025

Electronic publication

## References

[1] Kallingal A (2023). Cancer immune escape: the role of antigen presentation machinery. J Cancer Res Clin Oncol.

[2] Sade-Feldman M (2017). Resistance to checkpoint blockade therapy through inactivation of antigen presentation. Nat Commun.

[3] Gettinger S (2017). Impaired HLA class I antigen processing and presentation as a mechanism of acquired resistance to immune checkpoint inhibitors in lung cancer. Cancer Discov.

[4] Rock KL (2016). Present yourself! By MHC class I and MHC class II molecules. Trends Immunol.

[5] Laidlaw BJ (2016). The multifaceted role of CD4(+) T cells in CD8(+) T cell memory. Nat Rev Immunol.

[6] Sari G, Rock KL (2023). Tumor immune evasion through loss of MHC class-I antigen presentation. Curr Opin Immunol.

[7] Burr ML (2019). An evolutionarily conserved function of polycomb silences the MHC class I antigen presentation pathway and enables immune evasion in cancer. Cancer Cell.

[8] Chen XF (2023). A membrane-associated MHC-I inhibitory axis for cancer immune evasion. Cell.

[9] Chew GL (2019). DUX4 suppresses MHC class I to promote cancer immune evasion and resistance to checkpoint blockade. Dev Cell.

[10] Janssen EM (2003). CD4+ T cells are required for secondary expansion and memory in CD8+ T lymphocytes. Nature.

[11] Speiser DE (2023). CD4^+^ T cells in cancer. Nat Cancer.

[12] Cachot A (2021). Tumor-specific cytolytic CD4 T cells mediate immunity against human cancer. Sci Adv.

[13] Xie Y (2010). Naive tumor-specific CD4(+) T cells differentiated in vivo eradicate established melanoma. J Exp Med.

[14] Quezada SA (2010). Tumor-reactive CD4(+) T cells develop cytotoxic activity and eradicate large established melanoma after transfer into lymphopenic hosts. J Exp Med.

[15] Kreiter S (2015). Mutant MHC class II epitopes drive therapeutic immune responses to cancer. Nature.

[16] Oh DY (2020). Intratumoral CD4^+^ T cells mediate anti-tumor cytotoxicity in human bladder cancer. Cell.

[17] Steimle V (1994). Regulation of MHC class II expression by interferon-gamma mediated by the transactivator gene CIITA. Science.

[18] Doherty TM (1995). T-cell regulation of macrophage function. Curr Opin Immunol.

[19] Dembic Z (2000). Dendritic cells purified from myeloma are primed with tumor-specific antigen (idiotype) and activate CD4+ T cells. Proc Natl Acad Sci U S A.

[20] de Charette M (2016). Turning tumour cells into antigen presenting cells: the next step to improve cancer immunotherapy?. Eur J Cancer.

[21] Younger AR (2008). HLA class II antigen presentation by prostate cancer cells. Prostate Cancer Prostatic Dis.

[22] Oldford SA (2004). HLA-DRB alleles are differentially expressed by tumor cells in breast carcinoma. Int J Cancer.

[23] Forero A (2016). Expression of the MHC class II pathway in triple-negative breast cancer tumor cells is associated with a good prognosis and infiltrating lymphocytes. Cancer Immunol Res.

[24] Roemer MGM (2018). Major histocompatibility complex class II and programmed death ligand 1 expression predict outcome after programmed death 1 blockade in classic Hodgkin lymphoma. J Clin Oncol.

[25] Rodig SJ (2018). MHC proteins confer differential sensitivity to CTLA-4 and PD-1 blockade in untreated metastatic melanoma. Sci Transl Med.

[26] Callahan MJ (2008). Increased HLA-DMB expression in the tumor epithelium is associated with increased CTL infiltration and improved prognosis in advanced-stage serous ovarian cancer. Clin Cancer Res.

[27] Axelrod ML (2019). Biological consequences of MHC-II expression by tumor cells in cancer. Clin Cancer Res.

[28] Pérez-González A (2023). Cancer cell plasticity during tumor progression, metastasis and response to therapy. Nat Cancer.

[29] Chu X (2024). Cancer stem cells: advances in knowledge and implications for cancer therapy. Signal Transduct Target Ther.

[30] Takebe N (2015). Targeting Notch, Hedgehog, and Wnt pathways in cancer stem cells: clinical update. Nat Rev Clin Oncol.

[31] Gu Y (2023). Harnessing epithelial-mesenchymal plasticity to boost cancer immunotherapy. Cell Mol Immunol.

[32] Maksour S (2020). More than a corepressor: the role of CoREST proteins in neurodevelopment. eNeuro.

[33] Yang P (2011). RCOR2 is a subunit of the LSD1 complex that regulates ESC property and substitutes for SOX2 in reprogramming somatic cells to pluripotency. Stem Cells.

[34] Wang Y (2016). LSD1 co-repressor Rcor2 orchestrates neurogenesis in the developing mouse brain. Nat Commun.

[35] Barrios AP (2014). Differential properties of transcriptional complexes formed by the CoREST family. Mol Cell Biol.

[36] Song Y (2020). Mechanism of crosstalk between the LSD1 demethylase and HDAC1 deacetylase in the CoREST complex. Cell Rep.

[37] Heddleston JM (2010). Hypoxia inducible factors in cancer stem cells. Br J Cancer.

[38] Luo W, Wang Y (2019). Hypoxia mediates tumor malignancy and therapy resistance. Adv Exp Med Biol.

[39] Ortiz-Barahona A (2010). Genome-wide identification of hypoxia-inducible factor binding sites and target genes by a probabilistic model integrating transcription-profiling data and in silico binding site prediction. Nucleic Acids Res.

[40] Ryland GL (2013). RNF43 is a tumour suppressor gene mutated in mucinous tumours of the ovary. J Pathol.

[41] Chan JM (2024). Inherited BRCA1 and RNF43 pathogenic variants in a familial colorectal cancer type X family. Fam Cancer.

[42] Jiang X (2013). Inactivating mutations of RNF43 confer Wnt dependency in pancreatic ductal adenocarcinoma. Proc Natl Acad Sci U S A.

[43] Kasuga Y (2023). FBXO11 constitutes a major negative regulator of MHC class II through ubiquitin-dependent proteasomal degradation of CIITA. Proc Natl Acad Sci U S A.

[44] Ulbricht T (2012). PML promotes MHC class II gene expression by stabilizing the class II transactivator. J Cell Biol.

[45] Buxade M (2018). Macrophage-specific MHCII expression is regulated by a remote Ciita enhancer controlled by NFAT5. J Exp Med.

[46] Fan Z (2016). The arginine methyltransferase PRMT5 regulates CIITA-dependent MHC II transcription. Biochim Biophys Acta.

[47] Oliveira G (2022). Landscape of helper and regulatory antitumour CD4^+^ T cells in melanoma. Nature.

[48] Laumont CM, Nelson BH (2023). B cells in the tumor microenvironment: multi-faceted organizers, regulators, and effectors of anti-tumor immunity. Cancer Cell.

[49] Cortes J (2022). Pembrolizumab plus chemotherapy in advanced triple-negative breast cancer. N Engl J Med.

[50] Sun Q (2023). Immune checkpoint therapy for solid tumours: clinical dilemmas and future trends. Signal Transduct Target Ther.

[51] Lee J, Kim EH (2023). Mechanisms underlying response and resistance to immune checkpoint blockade in cancer immunotherapy. Front Oncol.

[52] Lorenzo-Sanz L (2024). Cancer cell plasticity defines response to immunotherapy in cutaneous squamous cell carcinoma. Nat Commun.

[53] Nadal E (2023). A phase Ib/II study of galunisertib in combination with nivolumab in solid tumors and non-small cell lung cancer. BMC Cancer.

[54] Doench JG (2016). Optimized sgRNA design to maximize activity and minimize off-target effects of CRISPR-Cas9. Nat Biotechnol.

[55] Bao L (2023). SAP30 promotes breast tumor progression by bridging the transcriptional corepressor SIN3 complex and MLL1. J Clin Invest.

[56] Luo M (2022). ZMYND8 is a master regulator of 27-hydroxycholesterol that promotes tumorigenicity of breast cancer stem cells. Sci Adv.

[57] Hu Y, Smyth GK (2009). ELDA: extreme limiting dilution analysis for comparing depleted and enriched populations in stem cell and other assays. J Immunol Methods.

