# Supplementary material for: Tumoral RCOR2 promotes tumor development through dual epigenetic regulation of tumor plasticity and immunogenicity
Source: J Clin Invest. 2025 Jul 3;135(18):e188801. doi: 10.1172/JCI188801 (PMC12435851; doi:10.1172/JCI188801)
Supplement: Supplemental data [file jci-135-188801-s176.pdf]

Supplemental Information

Tumoral RCOR2 promotes tumor development through dual epigenetic regulation of tumor plasticity and immunogenicity

Lei Bao[1], Ming Zhu[1], Maowu Luo[1], Ashwani Kumar[2], Yan Peng[1], Chao Xing[2,3,4], Yingfei Wang[1,5,6], Weibo Luo[1,7*]

[1]Department of Pathology,

[2]Eugene McDermott Center for Human Growth and Development,

[3]Lyda Hill Department of Bioinformatics,

[4]O'Donnell School of Public Health,

[5]Department of Neurology,

[6]Peter O'Donnell Jr. Brain Institute,

[7]Department of Pharmacology,

UT Southwestern Medical Center, Dallas, TX 75390, USA.

Supplemental Table 1. Oligonucleotide sequence used for sgRNAs

Target genes	Sequences
human *RCOR2* sgRNA1	5'-GAGTGGGGCTGTCCGCCGTT-3'
human *RCOR2* sgRNA2	5'-TCTGTGGCATAAGCACGATG-3'
human *RNF43* sgRNA1	5'-CTTCAGCTCAATCCTCACAT-3'
human *RNF43* sgRNA2	5'-TTACCCCAGATCAACACCAC-3'
human *HDAC1* sgRNA	5'-ACTCGTCATCAATCCCGTCT-3'
human *HDAC2* sgRNA	5'-TACAACAGATCGTGTAATGA-3'
mouse *Rcor2* sgRNA1	5'-CCCTGATGAGTGGACGGTGG-3'
mouse *Rcor2* sgRNA2	5'-CCATCACACTAGTTCGACTT-3'
mouse *Ciita* sgRNA	5'-ATGGATCTGGGATCTCCAGA-3'
mouse *H2-Ab1* sgRNA	5'-AGTGAGGAACCAATCAGCAT-3'
mouse *H2-Ea* sgRNA	5'-GGGATGGAAGAGCATAGAAT-3'

Supplemental Table 2. The list of antibodies used in this paper

Antibodies	Source	Catalog#
anti-RCOR2	Customized	N/A
anti-Actin	Proteintech	66009-1-Ig
anti-CIITA	Santa Cruz Biotechnology	SC-13556
anti-HLA-DR/DP/DQ/DX	Santa Cruz Biotechnology	SC-53302
anti-MHC Class II (I-A/I-E)	Thermo Fisher Scientific	14-5321-82
anti-phospho-β-Catenin (Ser33/37/Thr41)	Cell Signaling Technology	9561
anti-β-Catenin	Cell Signaling Technology	8480
anti-RNF43	Sigma-Aldrich	MABC606
anti-Histone H3	Cell Signaling Technology	4620
anti-HA-Tag	Cell Signaling Technology	3724
anti-LSD1	Cell Signaling Technology	2184
anti-HDAC1	Bethyl Laboratories	A300-713A
anti-HDAC2	Bethyl Laboratories	A300-705A
anti-CD4	Cell Signaling Technology	25229
anti-CD8α	Cell Signaling Technology	98941
anti-H3K4me2	Cell Signaling Technology	9725
anti-H4K16Ac	Cell Signaling Technology	13534
Normal rabbit IgG	Cell Signaling Technology	2729
anti-mCD45.2-PerCP-Cy5.5	BioLegend	109827
anti-mCD8a-Alexa Fluor 700	BioLegend	100730
anti-mCD4-Brilliant Violet 605	BioLegend	100548
anti-mCD3e-Brilliant Violet 786	BD Biosciences	564379
anti-mB220-Alexa Fluor 647	BioLegend	103226
anti-mFOXP3-PE	BioLegend	126403
anti-mGzmB-FITC	BioLegend	372206
anti-mCD11b-Brilliant Violet 421	BioLegend	101235
anti-mCD11c-FITC	BioLegend	117305
anti-mGr-1-Brilliant Violet 605	BioLegend	108439
anti-mF4/80-Brilliant Violet 785	BioLegend	123141
anti-mTER119-APC	BioLegend	116212
anti-mCD45-APC	BioLegend	103112
anti-mCD31-APC	BioLegend	102510
anti-mCD90.2-PE-Cy7	Invitrogen	25-0902-82
anti-mI-A/I-E-PerCP-Cy5.5	BioLegend	107625
anti-hCD24-FITC	BD Biosciences	555427
anti-hCD44-APC	BD Biosciences	559942
anti-hEpCAM-PerCP-eFluor 710	Invitrogen	46-3926-42

Supplemental Table 3. Oligonucleotide sequence used for qPCR primers

Genes	Primer sequence	Species
WNT5A	Forward: 5'-ATTCTTGGTGGTCGCTAGGTA-3'	human
	Reverse: 5'-CGCCTTCTCCGATGTACTGC-3'	
WNT10B	Forward: 5'-CATCCAGGCACGAATGCGA-3'	human
	Reverse: 5'-CGGTTGTGGGTATCAATGAAGA-3'	
RNF43	Forward: 5'-GGTGTTGATCTGGGGTAATGAC-3'	human
	Reverse: 5'-CAATCCTCACATGGGCCTTTT-3'	
CXXC4	Forward: 5'-ATGCACCACCGAAACGACTC-3'	human
	Reverse: 5'-GCAGTGTTCAGGGGATAAGGT-3'	
CIITA	Forward: 5'-AGACACCATCAACTGCGACC-3'	human
	Reverse: 5'-GCGATATTGGCATAAGCCTCC-3'	
HLA-DRA	Forward: 5'-AGTCCCTGTGCTAGGATTTTTCA-3'	human
	Reverse: 5'-ACATAAACTCGCCTGATTGGTC-3'	
HLA-DPB1	Forward: 5'-CAGCACCACAACCTGCTTG-3'	human
	Reverse: 5'-CCATTCAGGAACCATCGGACT-3'	
HLA-DMB	Forward: 5'-ACCTGTCTGTTGGATGATGCT-3'	human
	Reverse: 5'-CGCAAGGGGCCATCTTATTCT-3'	
Ciita	Forward: 5'-GGAGGAGATCGAACTCAGCTC-3'	mouse
	Reverse: 5'-GTTCCGCAATGTTGGCATAGG-3'	
H2-Aa	Forward: 5'-TGGGAGTCTTGACTAAGAGGTC-3'	mouse
	Reverse: 5'-CTGACTTGCTATTTCTGAGCCAT-3'	
H2-D	Forward: 5'-AGTGGTGCTGCAGAGCATTACAA-3'	mouse
	Reverse: 5'-GGTGACTTCACCTTTAGATCTGGG-3'	
H2-K	Forward: 5'-GCTGGTGAAGCAGAGAGACTCAG-3'	mouse
	Reverse: 5'-GGTGACTTTATCTTCAGGTCTGCT-3'	
Ifng	Forward: 5'-GCCACGGCACAGTCATTGA-3'	mouse
	Reverse: 5'-TGCTGATGGCCTGATTGTCTT-3'	
Il2	Forward: 5'-TGAGCAGGATGGAGAATTACAGG-3'	mouse
	Reverse: 5'-GTCCAAGTTCATCTTCTAGGCAC-3'	
Tnf	Forward: 5'-CAGGCGGTGCCTATGTCTC	mouse
	Reverse: 5'-CGATCACCCCGAAGTTCAGTAG-3'	
18S rRNA	Forward: 5'-CGGCGACGACCCATTCGAAC	human/mouse
	Reverse: 5'-GAATCGAACCCTGATTCCCCGTC-3'	

Supplemental Table 4. Oligonucleotide sequence used for ChIP-qPCR primers

Genes	Primer sequence	Species
RNF43	Forward: 5’-GAAGCAGTTTAGGAGGTGTATGT-3’	human
	Reverse: 5’-CTGTTTCATCTTGAGGGTCGT-3’	
CIITA	Forward: 5’-GACCAAAGTCTCCTCTGTAACC-3’	human
	Reverse: 5’-CTGTGTGACCTTGAGCAAGTA-3’	

Supplemental Table 5. Oligonucleotide sequence used for mouse genotyping

Targets	Primer sequence
Floxed *Rcor2*	Forward: 5’-CTCACACTCCGAGGTCTTGA-3’
	Reverse: 5’-CAGGCTTGACACTGCACCA-3’
MMTV-PyMT	Forward: 5’-GGAAGCAAGTACTTCACAAGGG-3’
	Reverse: 5’-GGAAAGTCACTAGGAGCAGGG-3’
MMTV-PyMT internal control	Forward: 5’-CAAATGTTGCTTGTCTGGTG-3’
	Reverse: 5’-GTCAGTCGAGTGCACAGTTT-3’
K14-Cre	Forward: 5’-TTCCTCAGGAGTGTCTTCGC-3’
	Reverse: 5’-GTCCATGTCCTTCCTGAAGC-3’
K14-Cre internal control	Forward: 5’-CTAGGCCACAGAATTGAAAGATCT-3’
	Reverse: 5’-GTAGGTGGAAATTCTAGCATCATCC-3’

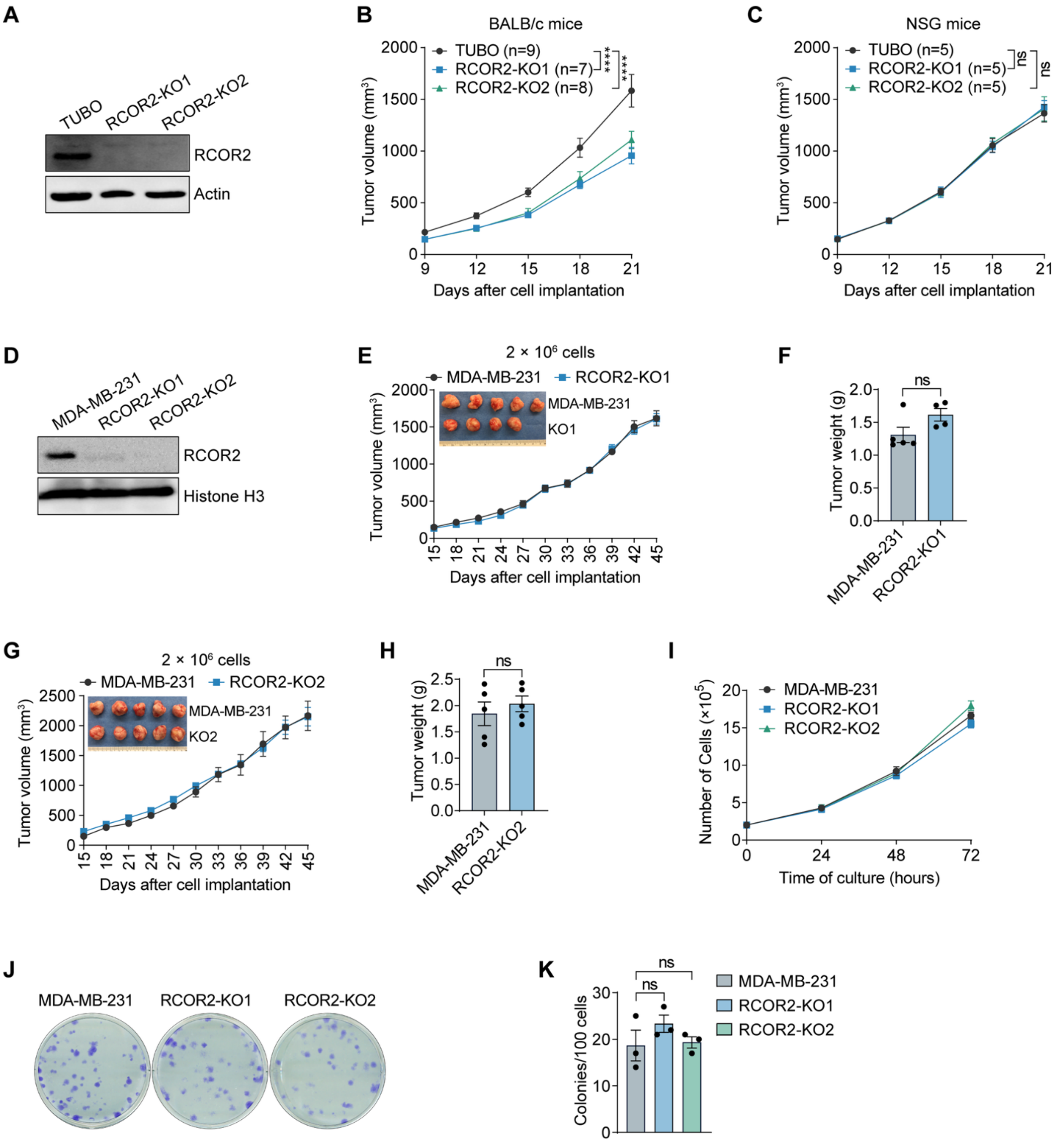

Figure S1. RCOR2 promotes immune evasion in breast tumors.

(**A**) Immunoblot analysis of RCOR2 protein in parental and RCOR2-KO1 or -KO2 TUBO cells.

(**B** and **C**) Growth of parental and RCOR2-KO1 or -KO2 TUBO tumors in BALB/c (**B**) and NSG (**C**) mice (mean ± SEM). ****$P < 0.0001$, by two-way ANOVA with Dunnett's test. ns, not significant.

(**D**) Immunoblot analysis of RCOR2 protein in parental and RCOR2-KO1 or -KO2 MDA-MB-231 cells.

(**E**-**H**) Growth of parental and RCOR2-KO1 (**E**) or -KO2 (**G**) MDA-MB-231 tumors in NSG mice. After harvesting, tumors were imaged (inset) and weighed (**F** and **H**). mean ± SEM, n = 4-5.

(**I**) Proliferation assay of parental and RCOR2-KO1 or -KO2 MDA-MB-231 cells (mean ± SEM, n = 3).

(**J** and **K**) Colony formation of parental and RCOR2-KO1 or -KO2 MDA-MB-231 cells. Representative images are shown in **J**. Quantification of colony numbers is shown in **K** (mean ± SEM, n = 3).

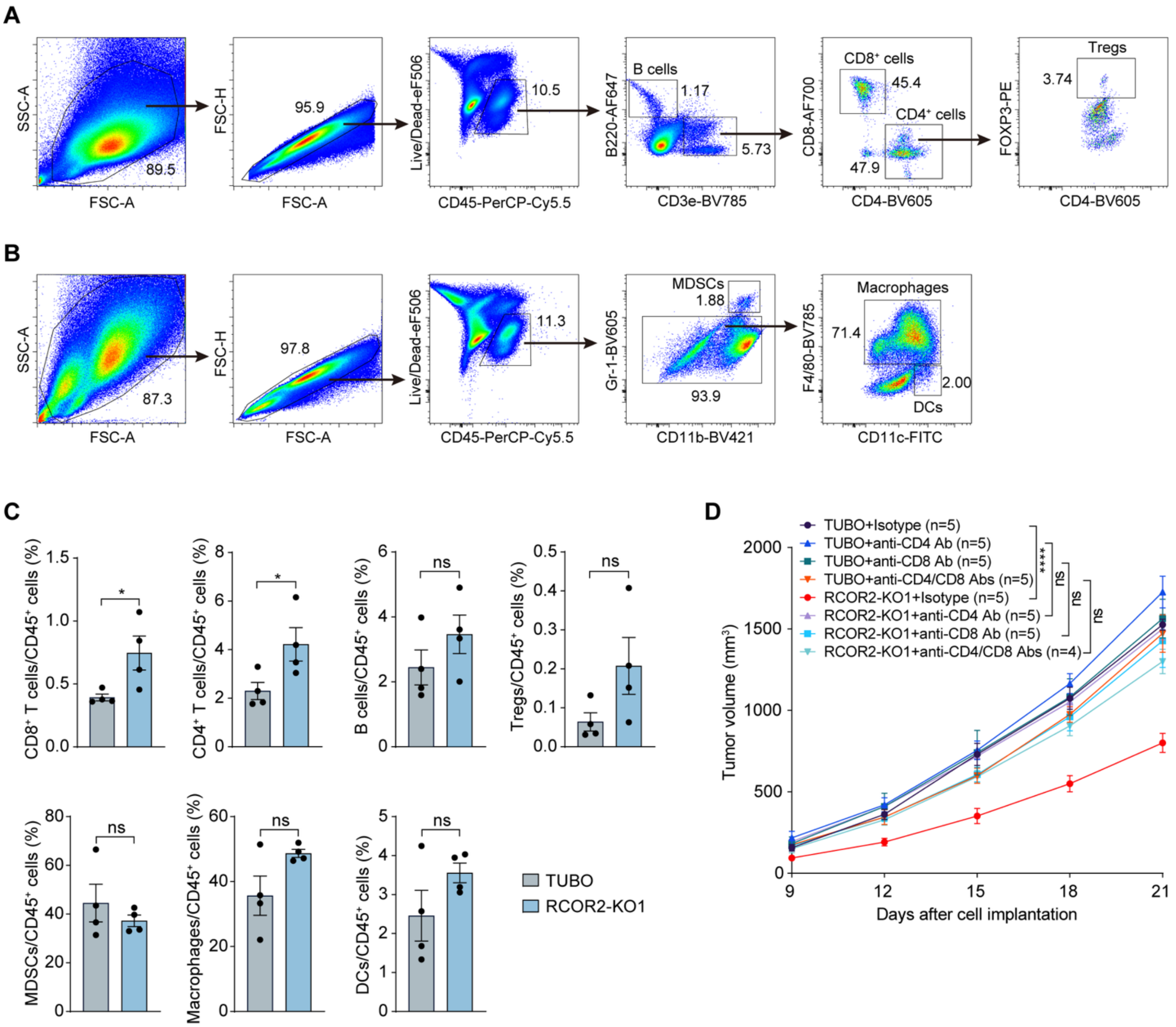

Figure S2. Tumoral RCOR2 shapes the lymphocyte landscape in breast tumors.

(**A** and **B**) Representative gating of lymphocytes (**A**) and myeloid cells (**B**) by flow cytometry.

(**C**) Flow cytometry analysis of $CD8^+$ T cells ($CD45^+CD3e^+CD8^+$), $CD4^+$ T cells ($CD45^+CD3e^+CD4^+$), B cells ($CD45^+B220^+$), regulatory T cells ($CD45^+CD3e^+CD4^+FOXP3^+$), myeloid-derived suppressor cells ($CD45^+CD11b^+Gr\text{-}1^+$), macrophages ($CD45^+CD11b^+F4/80^+$) and dendritic cells ($CD45^+CD11c^+F4/80^-$) in parental and RCOR2-KO1 TUBO tumors (mean ± SEM, $n = 4$). *$P < 0.05$, by two-tailed Student's t test. ns, not significant.

(**D**) Growth of parental and RCOR2-KO1 TUBO tumors in BALB/c mice treated with IgG or anti-CD4 and/or anti-CD8 neutralizing antibodies (mean ± SEM). ****$P < 0.0001$, by two-way ANOVA with Tukey's test.

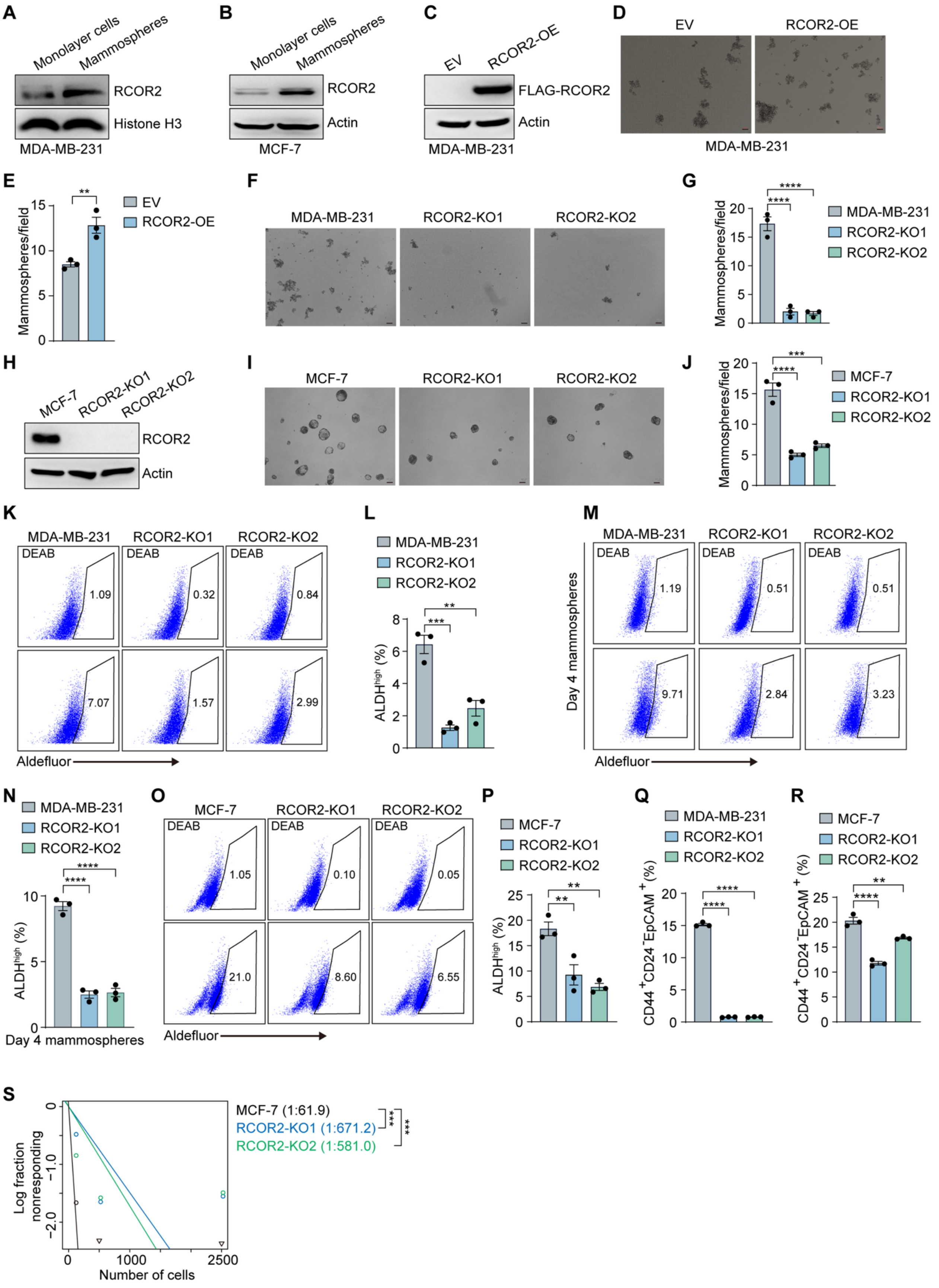
A
Monolayer cells
Mammospheres
RCOR2
Histone H3
MDA-MB-231
B
Monolayer cells
Mammospheres
RCOR2
Actin
MCF-7
C
EV
RCOR2-OE
FLAG-RCOR2
Actin
MDA-MB-231
D
EV
RCOR2-OE
MDA-MB-231
E
Mammospheres/field
EV
RCOR2-OE
F
MDA-MB-231
RCOR2-KO1
RCOR2-KO2
G
Mammospheres/field
MDA-MB-231
RCOR2-KO1
RCOR2-KO2
H
MCF-7
RCOR2-KO1
RCOR2-KO2
RCOR2
Actin
I
MCF-7
RCOR2-KO1
RCOR2-KO2
J
Mammospheres/field
MCF-7
RCOR2-KO1
RCOR2-KO2
K
MDA-MB-231
RCOR2-KO1
RCOR2-KO2
DEAB
1.09
0.32
0.84
7.07
1.57
2.99
Aldefluor
L
MDA-MB-231
RCOR2-KO1
RCOR2-KO2
ALDH^high (%)
M
MDA-MB-231
RCOR2-KO1
RCOR2-KO2
Day 4 mammospheres
DEAB
1.19
0.51
0.51
9.71
2.84
3.23
Aldefluor
N
MDA-MB-231
RCOR2-KO1
RCOR2-KO2
ALDH^high (%)
Day 4 mammospheres
O
MCF-7
RCOR2-KO1
RCOR2-KO2
DEAB
1.05
0.10
0.05
21.0
8.60
6.55
Aldefluor
P
MCF-7
RCOR2-KO1
RCOR2-KO2
ALDH^high (%)
Q
MDA-MB-231
RCOR2-KO1
RCOR2-KO2
CD44+CD24−EpCAM+ (%)
R
MCF-7
RCOR2-KO1
RCOR2-KO2
CD44+CD24−EpCAM+ (%)
S
Log fraction nonresponding
Number of cells
MCF-7 (1:61.9)
RCOR2-KO1 (1:671.2)
RCOR2-KO2 (1:581.0)

Figure S3. RCOR2 increases BCSC stemness and promotes tumor initiation.

(**A** and **B**) Immunoblot analysis of RCOR2 protein in MDA-MB-231 (**A**) and MCF-7 (**B**) monolayers and mammospheres.

(**C**) Immunoblot analysis of RCOR2 protein in RCOR2-overexpressing MDA-MB-231 cells.

(**D** and **E**) Mammosphere formation assay of control and RCOR2-overexpressing MDA-MB-231 cells. Representative mammosphere images are shown (**D**) and mammosphere numbers are quantified (**E**, mean ± SEM, n = 3). **P < 0.01, by two-tailed Student's t test. Scale bar, 100 μm.

(**F** and **G**) Mammosphere formation assay of parental and RCOR2-KO1 or -KO2 MDA-MB-231 cells. Representative mammosphere images are shown (**F**) and mammosphere numbers are quantified (**G**, mean ± SEM, n = 3). ****P < 0.0001, by one-way ANOVA with Dunnett's test. Scale bar, 100 μm.

(**H**) Immunoblot analysis of RCOR2 protein in parental and RCOR2-KO1 or -KO2 MCF-7 cells.

(**I** and **J**) Mammosphere formation assay in parental and RCOR2-KO1 or -KO2 MCF-7 cells. Representative mammosphere images are shown (**I**) and mammosphere numbers are quantified (**J**, mean ± SEM, n = 3). ***P < 0.001; ****P < 0.0001, by one-way ANOVA with Dunnett's test. Scale bar, 100 μm.

(**K** and **L**) Aldefluor assay in parental and RCOR2-KO1 or -KO2 MDA-MB-231 cells. Representative gating is shown (**K**) and $ALDH^{high}$ cells are quantified (**L**, mean ± SEM, n = 3). **P < 0.01; ***P < 0.001, by one-way ANOVA with Dunnett's test.

(**M** and **N**) Aldefluor assay in parental and RCOR2-KO1 or -KO2 MDA-MB-231 mammospheres. Representative gating is shown (**M**) and $ALDH^{high}$ cells are quantified (**N**, mean ± SEM, n = 3). ****P < 0.0001, by one-way ANOVA with Dunnett's test.

(**O** and **P**) Aldefluor assay in parental and RCOR2-KO1 or -KO2 MCF-7 cells. Representative gating is shown (**O**) and $ALDH^{high}$ cells are quantified (**P**, mean ± SEM, n = 3). **P < 0.01, by one-way ANOVA with Dunnett's test.

(**Q** and **R**) Flow cytometry analysis of $CD44^{+}CD24^{-}EpCAM^{+}$ BCSCs in parental and RCOR2-KO1 or -KO2 MDA-MB-231 (**Q**) and MCF-7 (**R**) cells (mean ± SEM, n = 3). **P < 0.01; ****P < 0.0001, by one-way ANOVA with Dunnett's test.

(**S**) Limiting dilution assay of parental and RCOR2-KO1 or -KO2 MCF-7 cells in NSG mice. ***P < 0.001, by χ^2 test.

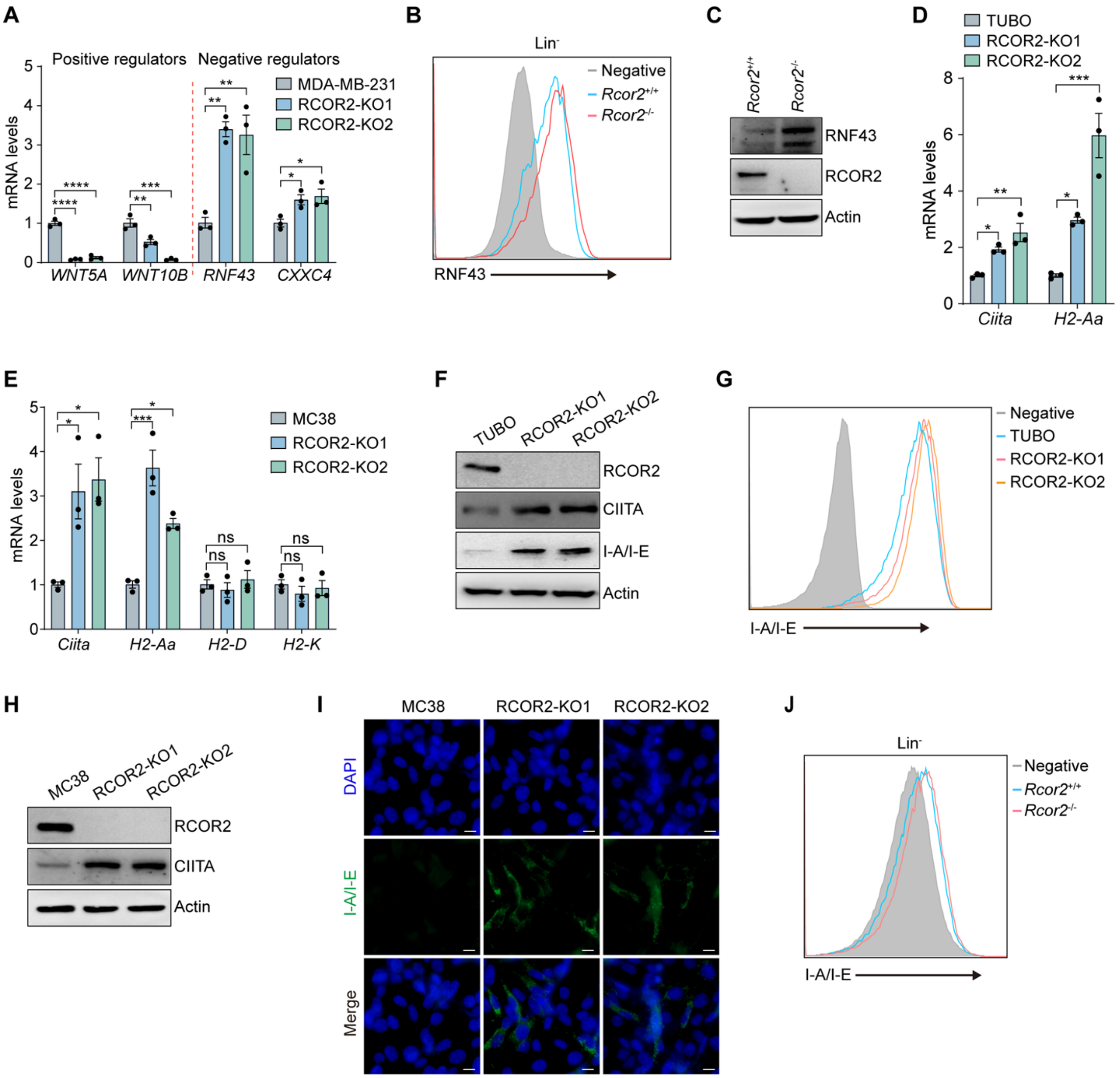

Figure S4. RCOR2 induces silencing of RNF43, CIITA, and MHC-II in cancer cells.

(**A**) RT-qPCR analysis of indicated mRNAs in parental, RCOR2-KO1 or -KO2 MDA-MB-231 cells (mean ± SEM, $n = 3$). *$P < 0.05$; **$P < 0.01$; ***$P < 0.001$; ****$P < 0.0001$, by one-way ANOVA with Dunnett's test.

(**B**) Flow cytometry analysis of RNF43 protein in Lin$^-$ cells from *MMTV-PyMT*$^{+/-}$;*K14-Cre*$^{+/-}$;*Rcor2*$^{+/+}$ and *MMTV-PyMT*$^{+/-}$;*K14-Cre*$^{+/-}$;*Rcor2*$^{fl/fl}$ tumors ($n = 3$).

(**C**) Immunoblot analysis of RNF43 and RCOR2 in *MMTV-PyMT*$^{+/-}$;*K14-Cre*$^{+/-}$;*Rcor2*$^{+/+}$ and *MMTV-PyMT*$^{+/-}$;*K14-Cre*$^{+/-}$;*Rcor2*$^{fl/fl}$ tumor cells.

(**D**) RT-qPCR analysis of indicated mRNAs in parental, RCOR2-KO1 or -KO2 TUBO cells treated with 1 ng/mL IFN-γ for 24 hours (mean ± SEM, $n = 3$). *$P < 0.05$; **$P < 0.01$; ***$P < 0.001$, by one-way ANOVA with Dunnett's test.

(**E**) RT-qPCR analysis of indicated mRNAs in parental, RCOR2-KO1 or -KO2 MC38 cells treated with 5 ng/mL IFN-γ for 24 hours (mean ± SEM, $n = 3$). *$P < 0.05$; ***$P < 0.001$, by one-way ANOVA with Dunnett's test. ns, not significant.

(**F**) Immunoblot analysis of indicated proteins in parental, RCOR2-KO1 or -KO2 TUBO cells treated with 1 ng/mL IFN-γ for 24 hours.

(**G**) Flow cytometry analysis of I-A/I-E protein in parental and RCOR2-KO1 or -KO2 TUBO cells treated with 1 ng/mL IFN-γ for 24 hours.

(**H**) Immunoblot analysis of CIITA in parental, RCOR2-KO1 or -KO2 MC38 cells treated with 5 ng/mL IFN-γ for 24 hours.

(**I**) Immunostaining of I-A/I-E in parental, RCOR2-KO1 or -KO2 MC38 cells treated with 5 ng/mL IFN-γ for 24 hours. Scale bar, 10 μm.

(**J**) Flow cytometry analysis of I-A/I-E protein in *MMTV-PyMT*$^{+/-}$;*K14-Cre*$^{+/-}$;*Rcor2*$^{+/+}$ and *MMTV-PyMT*$^{+/-}$;*K14-Cre*$^{+/-}$;*Rcor2*$^{fl/fl}$ tumor cells ($n = 3$).

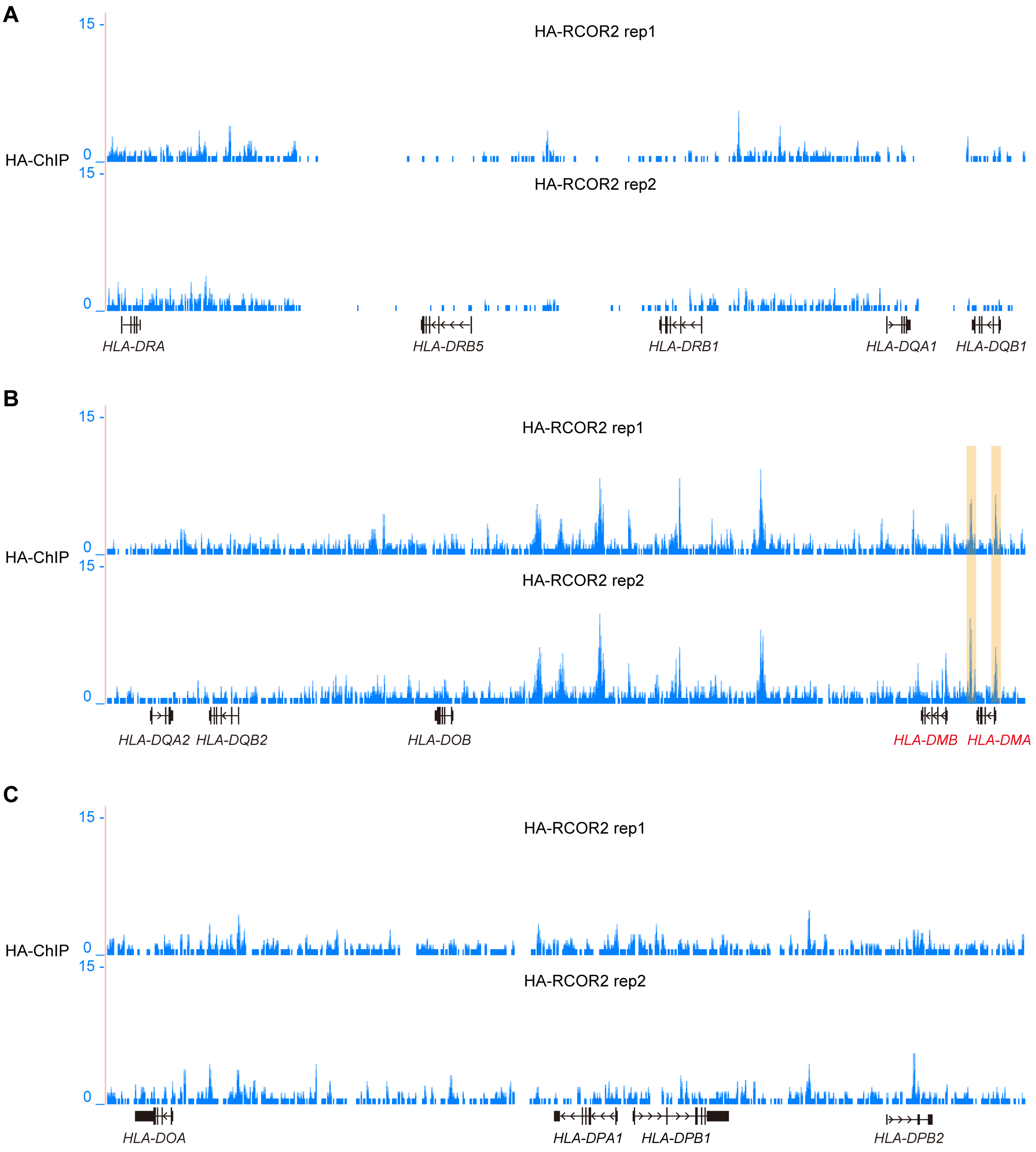

Figure S5. Enrichment of RCOR2 on MHC-II heavy chain genes in MDA-MB-231 cells.

(**A-C**) Genome browser snapshots of HA-RCOR2 ChIP-seq peaks on MHC-II heavy chain genes in MDA-MB-231 cells (*n* = 2). The RCOR2 binding peaks are highlighted in gold yellow.
