# Supplementary material for: Tumoral RCOR2 promotes tumor development through dual epigenetic regulation of tumor plasticity and immunogenicity
Source: J Clin Invest. 2025 Jul 3;135(18):e188801. doi: 10.1172/JCI188801 (PMC12435851; doi:10.1172/JCI188801)
Supplement: Unedited blot and gel images [file jci-135-188801-s177.pdf]

# Full unedited gel for Figure 1D

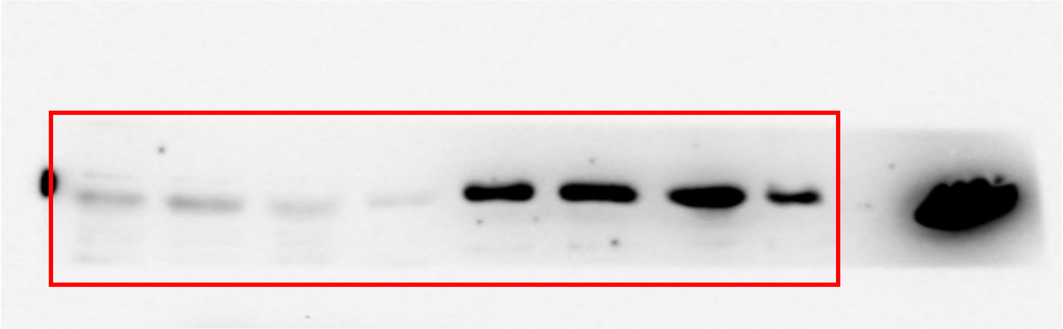

RCOR2

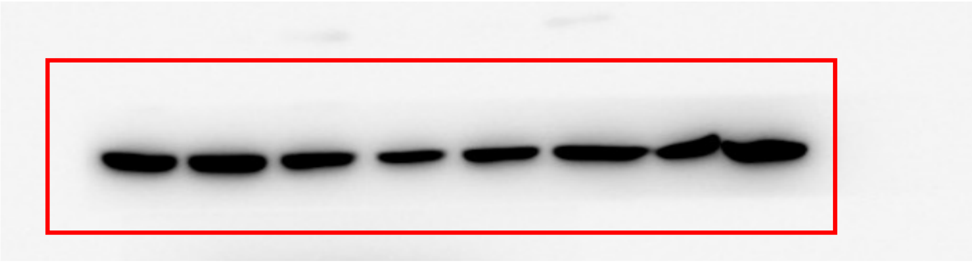

Actin

# Full unedited gel for Figure 2

A

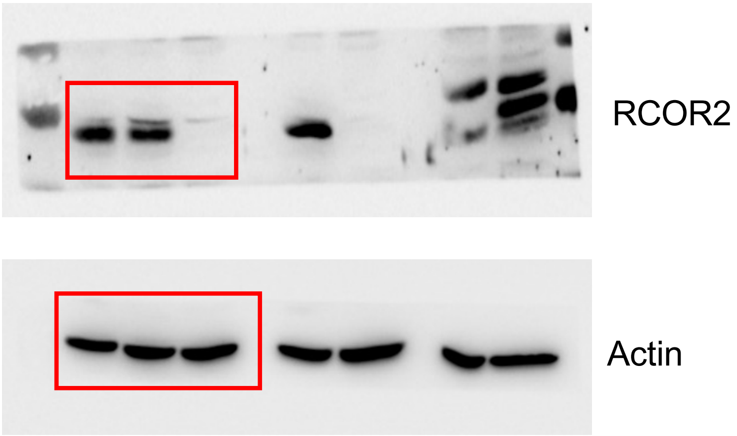


C

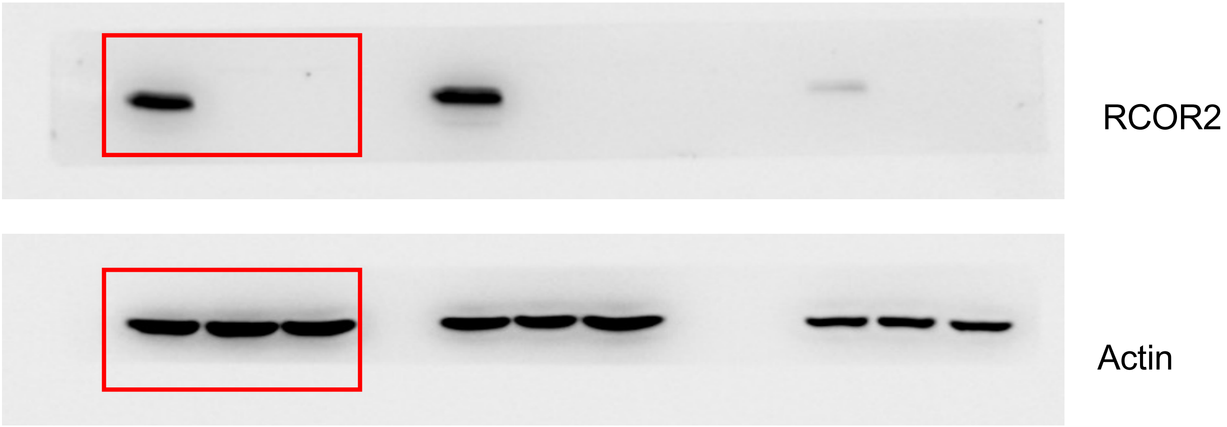

# Full unedited gel for Figure 3

C

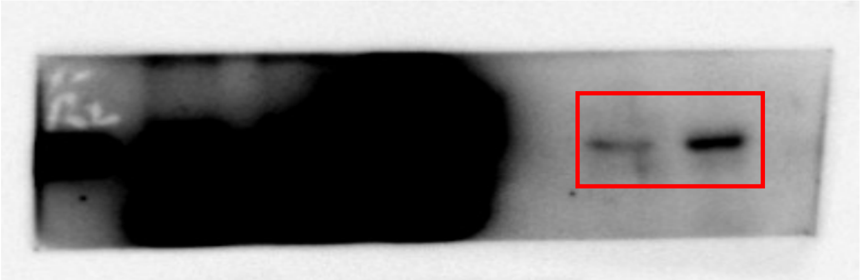

RCOR2

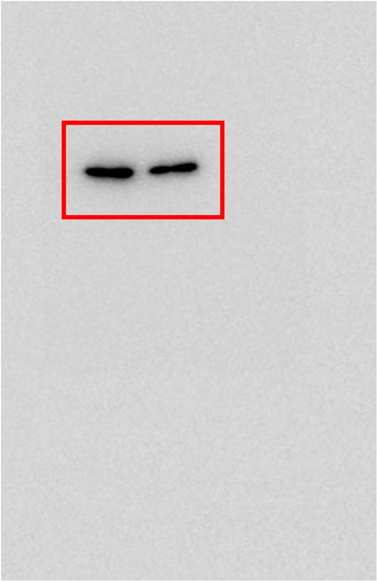

Actin

H

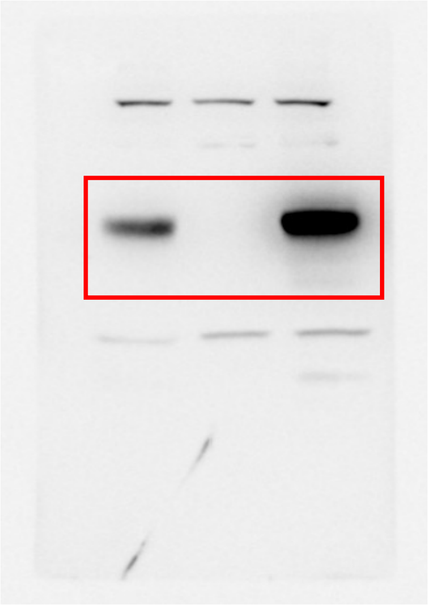

RCOR2

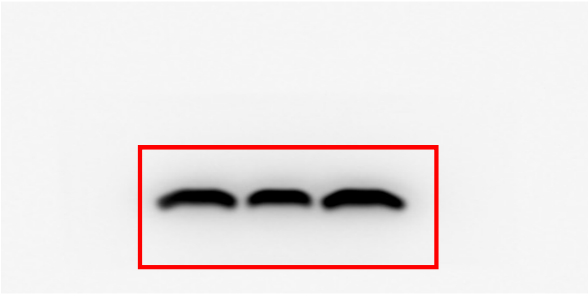

Histone H3

# Full unedited gel for Figure 4

J

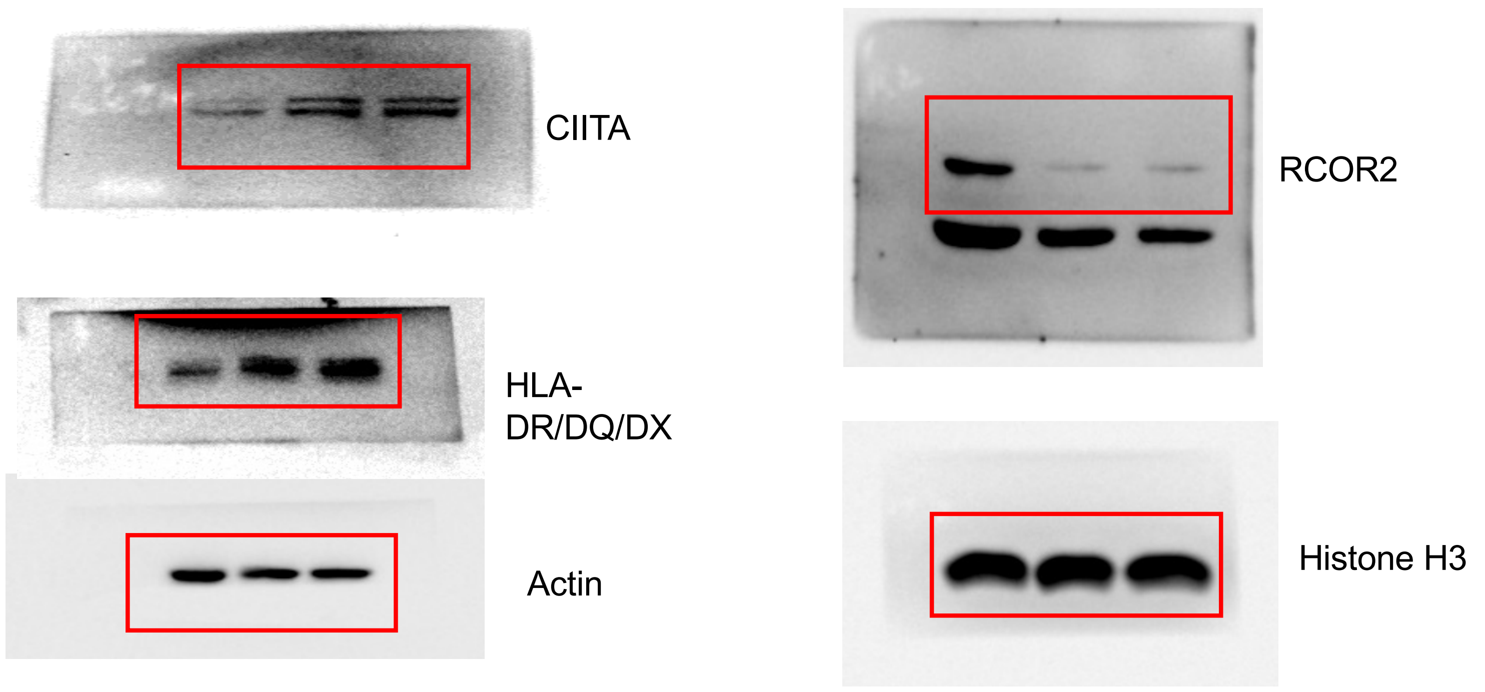


K

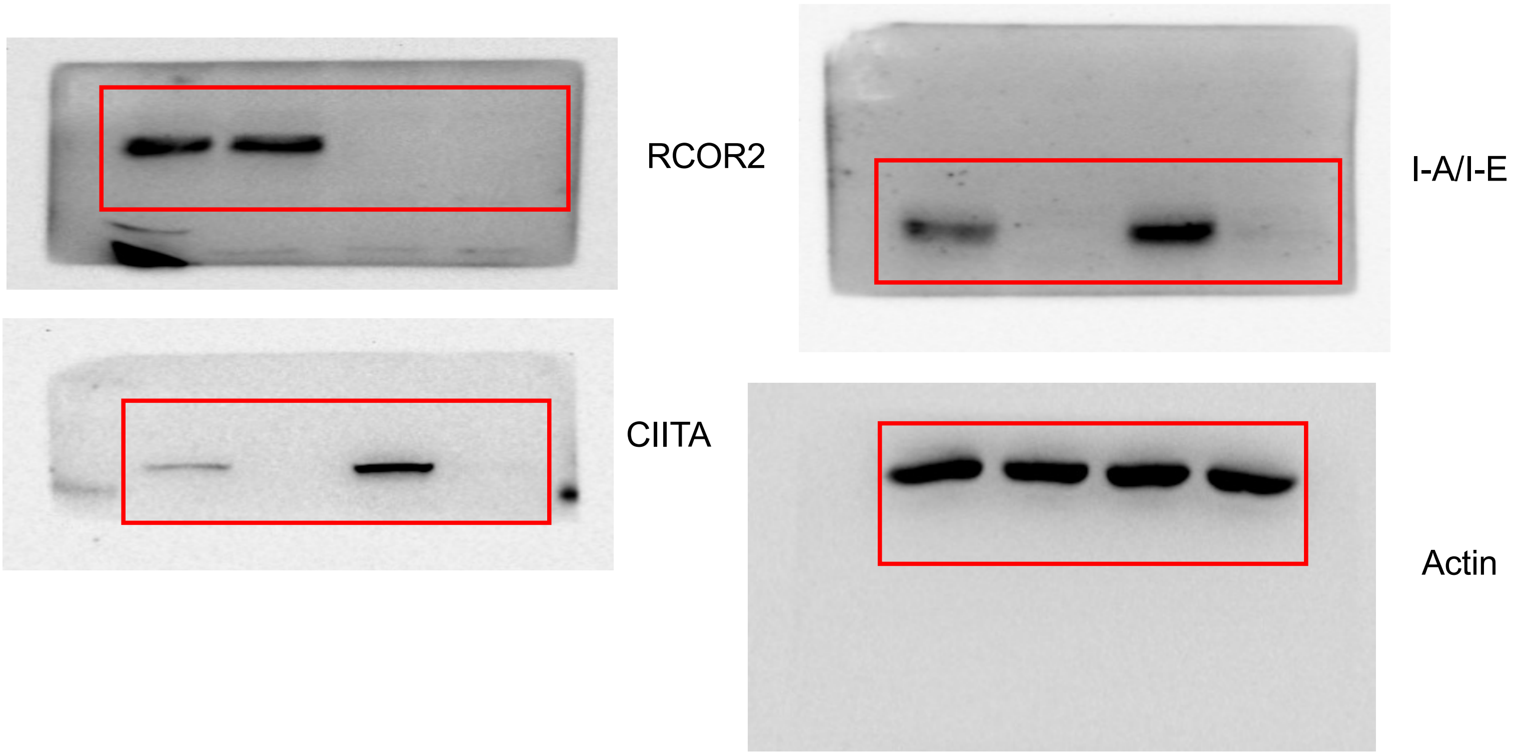

# Full unedited gel for Figure 4

L

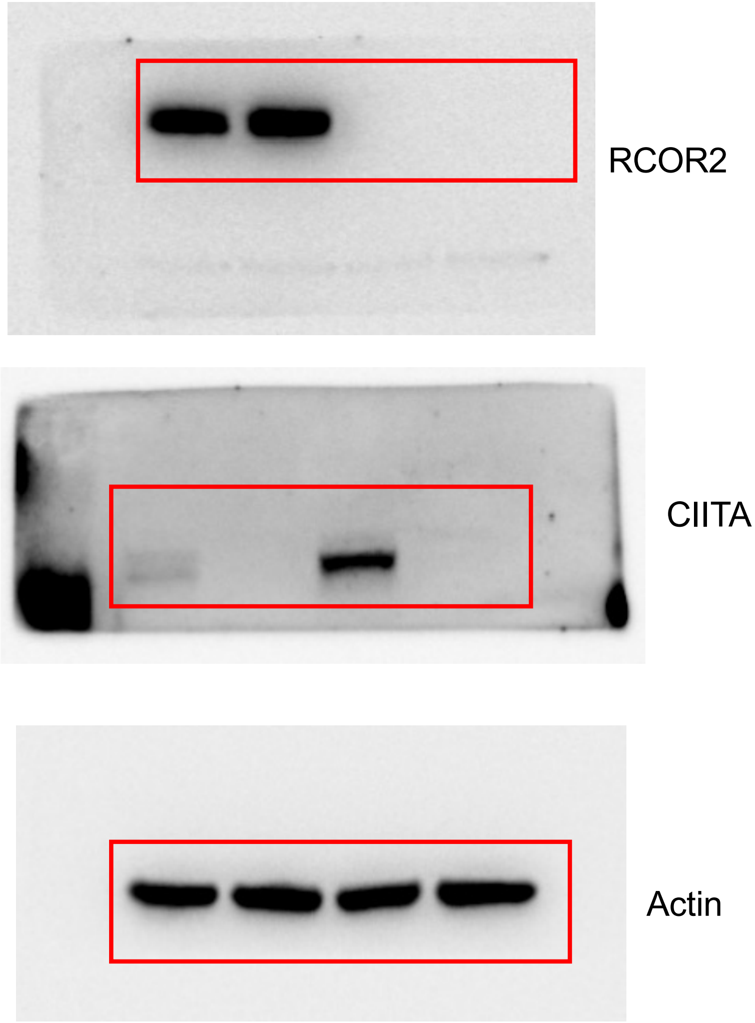

# Full unedited gel for Figure 5

D

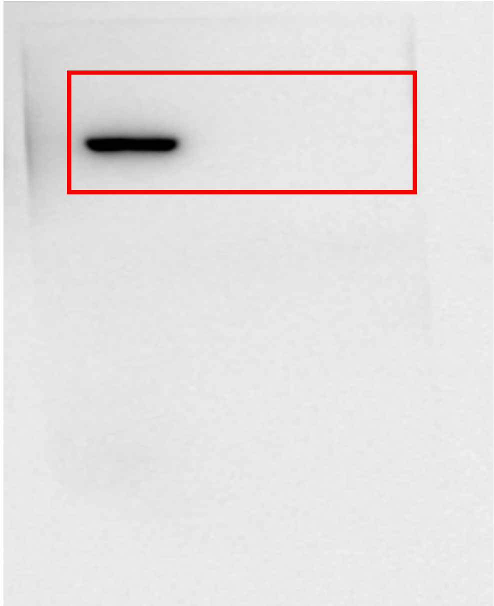

LSD1

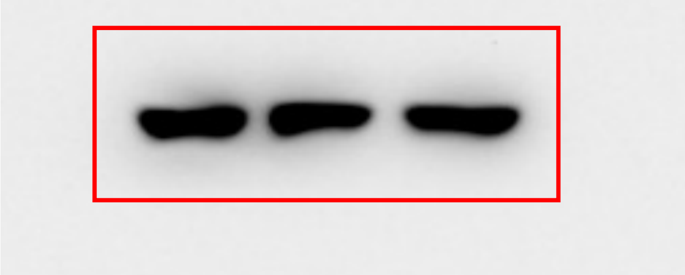

Actin

E

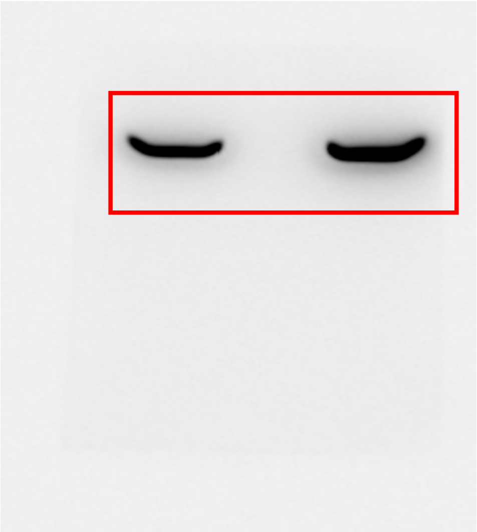

HDAC1

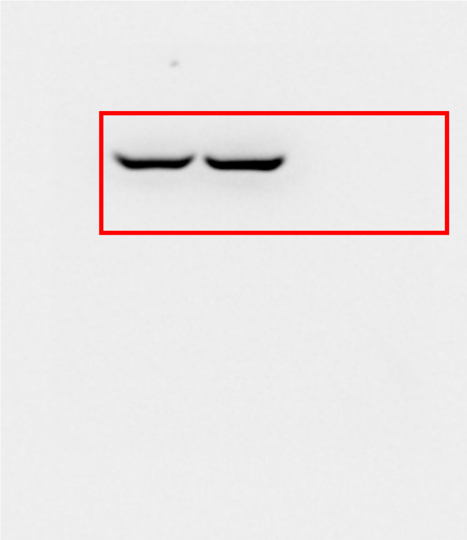

HDAC2

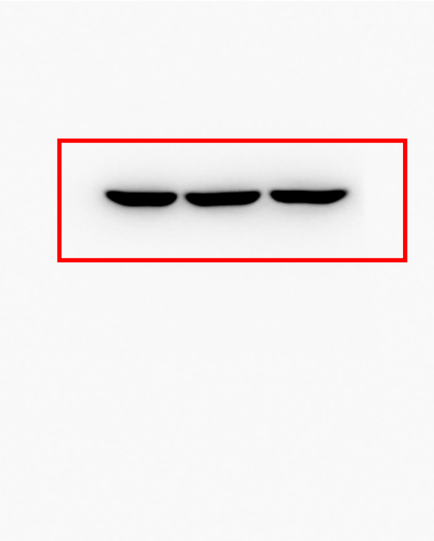

Actin

Full unedited gel for Figure 6

G

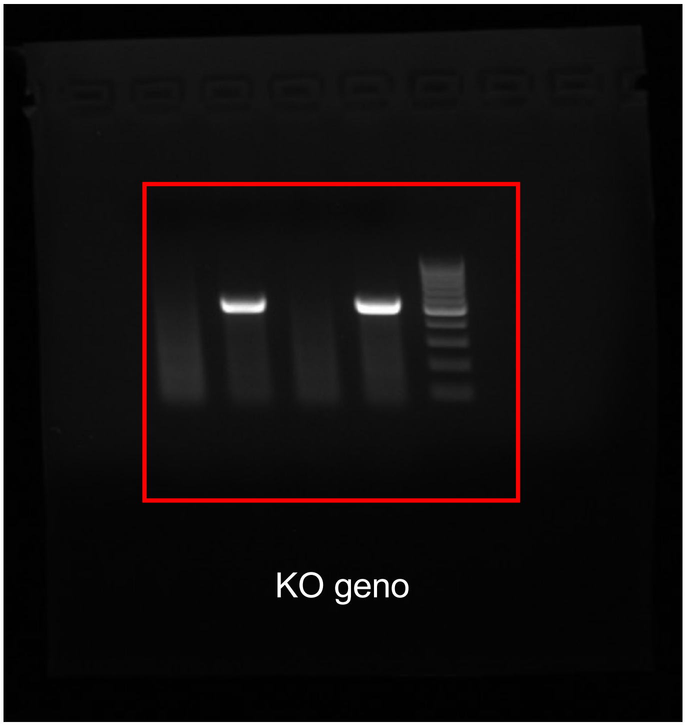


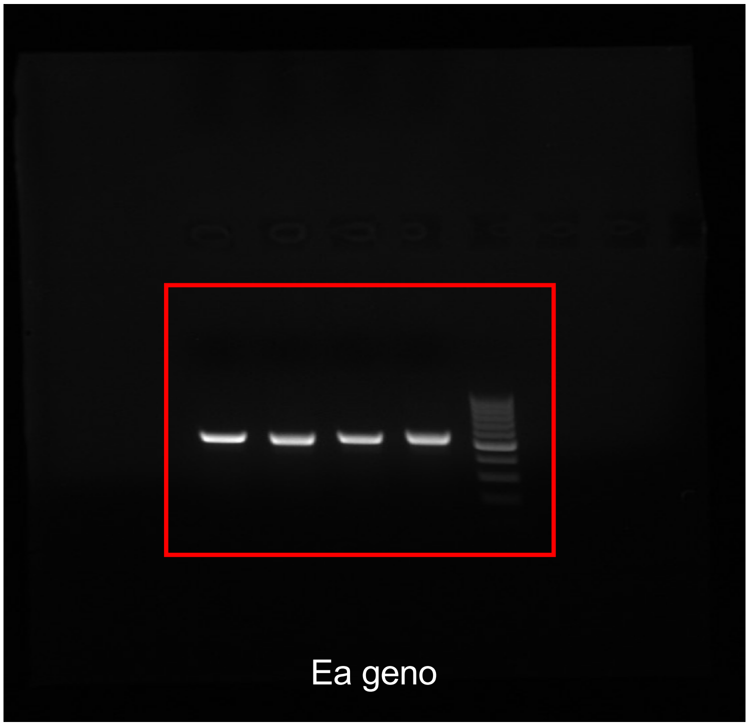


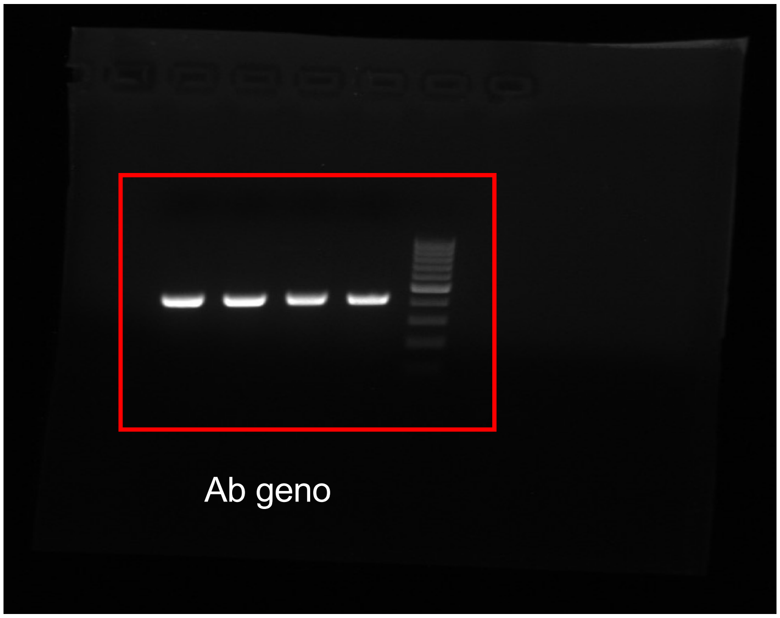

# Full unedited gel for Figure 8

A

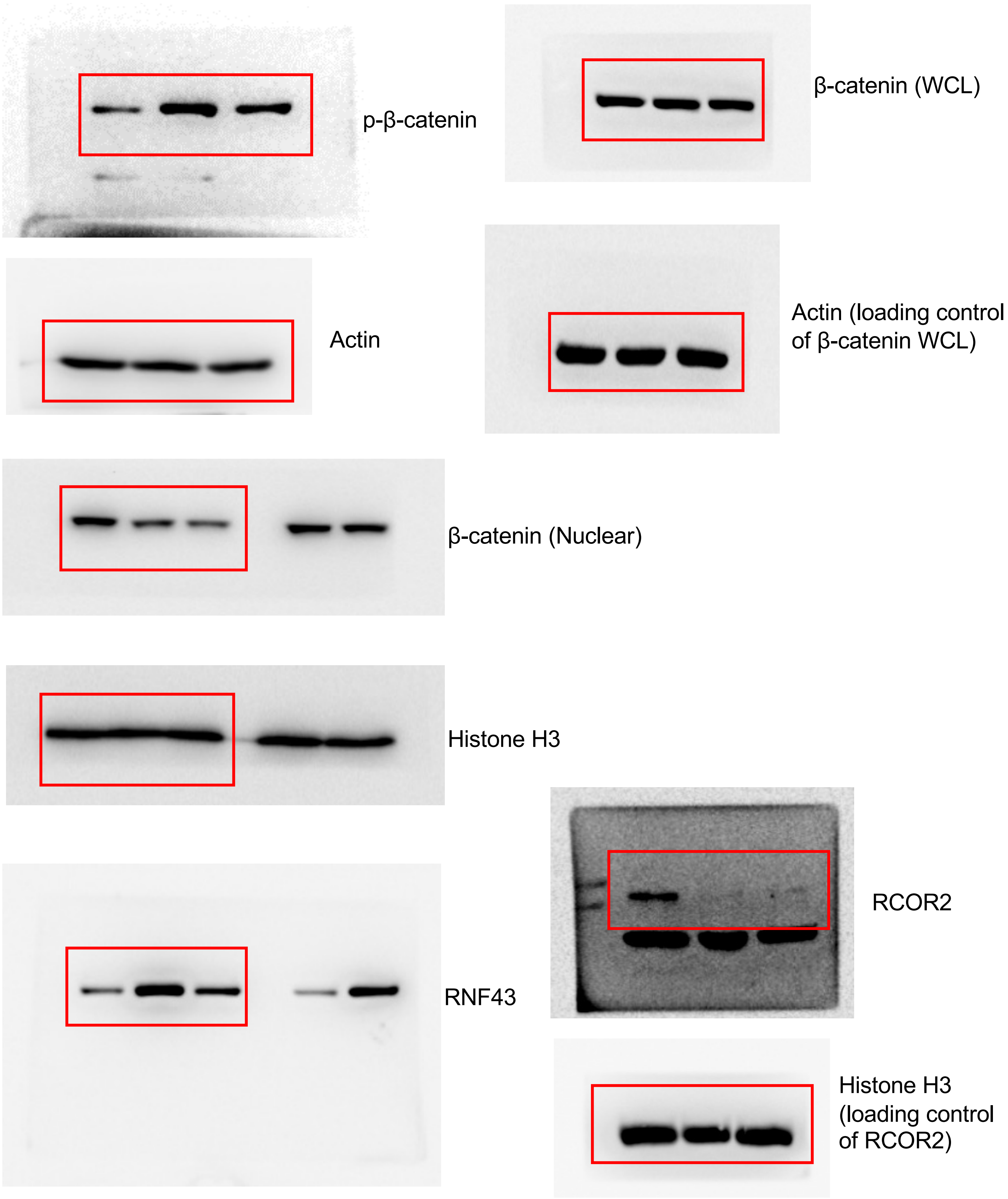

# Full unedited gel for Figure 8

B

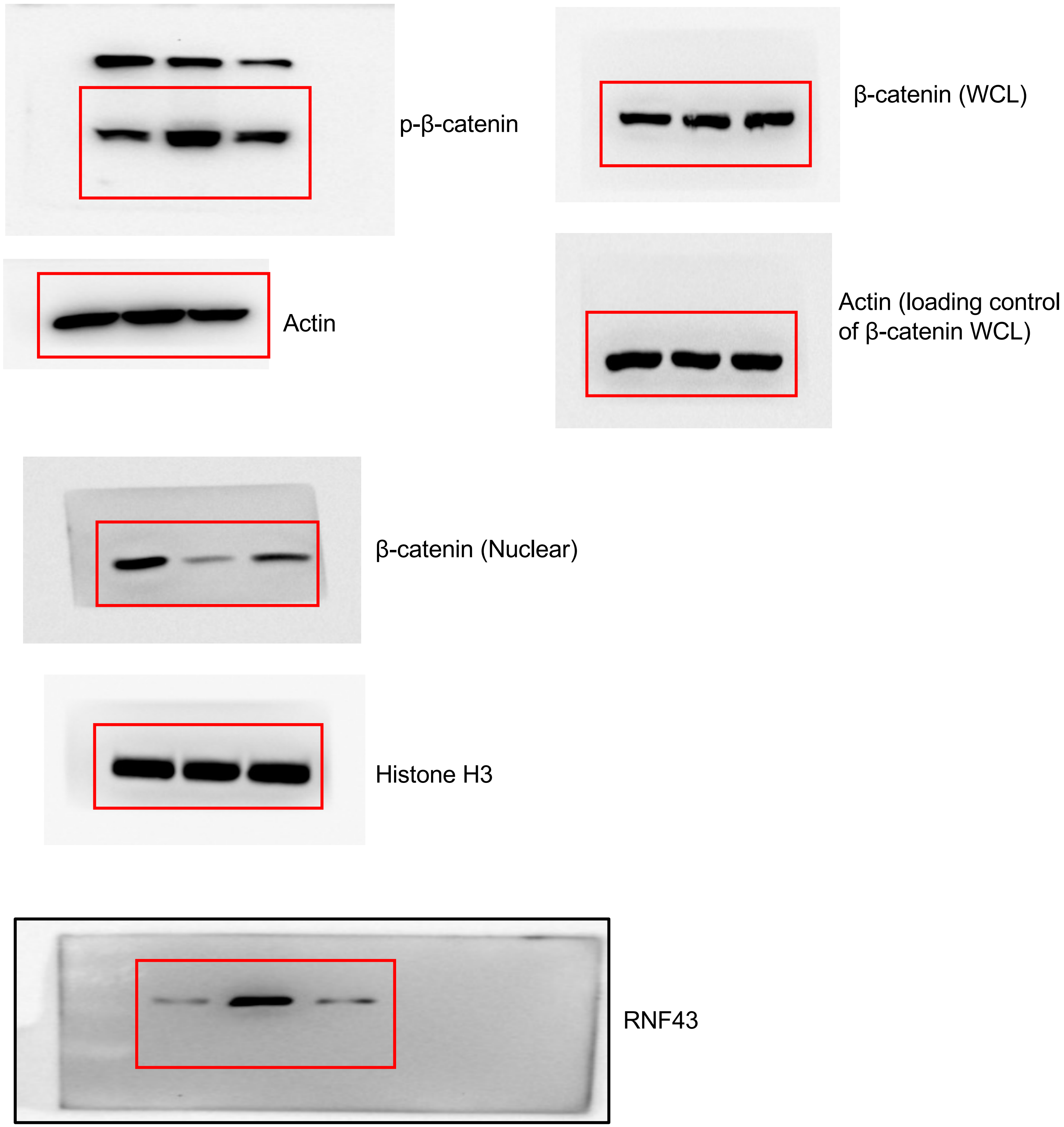

# Full unedited gel for Figure 8

C

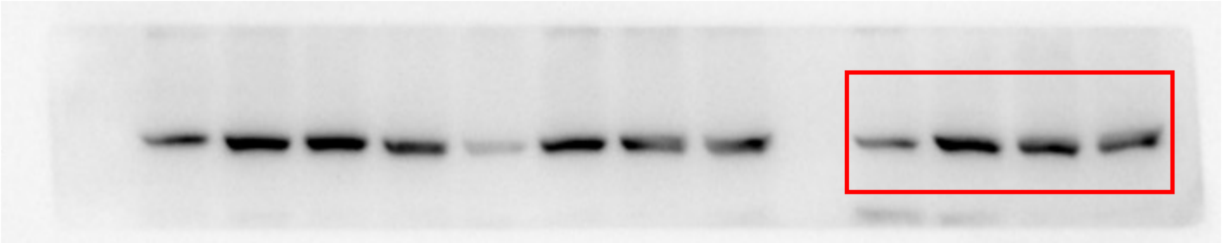

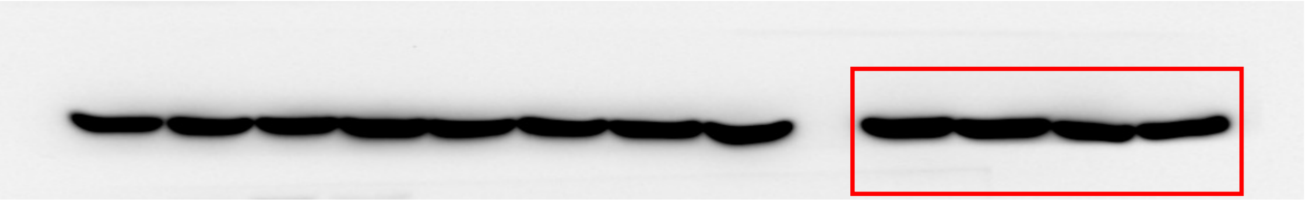

Actin

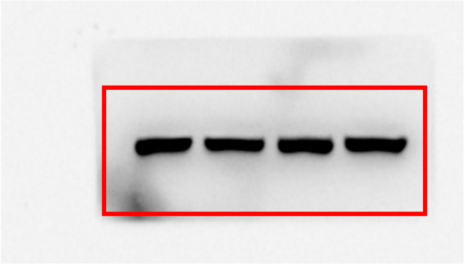

β-catenin (WCL)

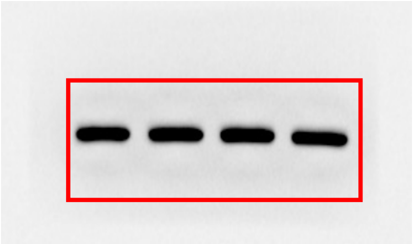

Actin (loading control of β-catenin WCL)

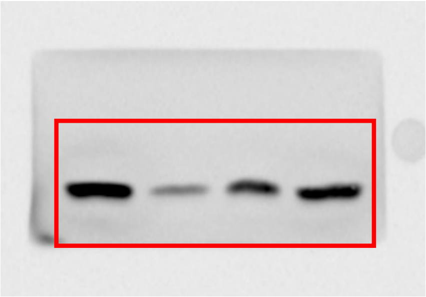

β-catenin (Nuclear)

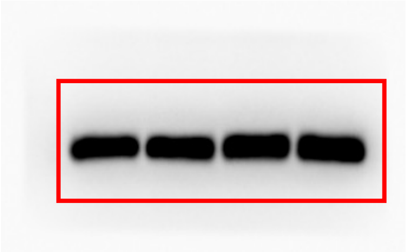

Histone H3

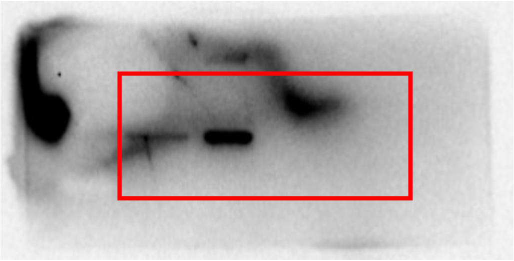

RNF43

# Full unedited gel for Figure 8

I

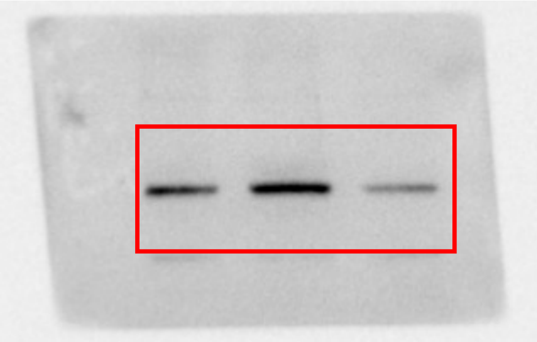

p-β-catenin

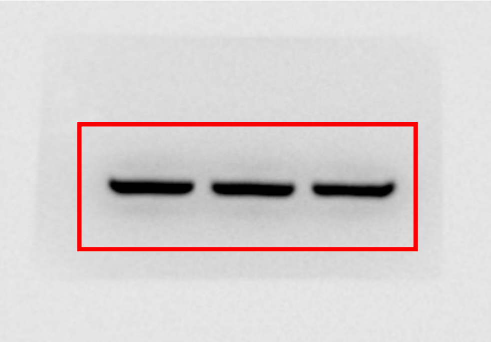

β-catenin

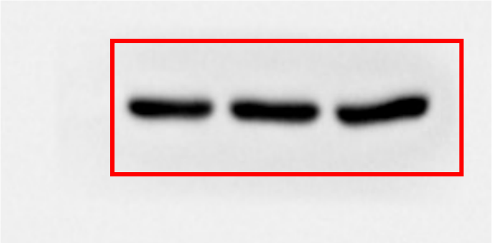

Actin

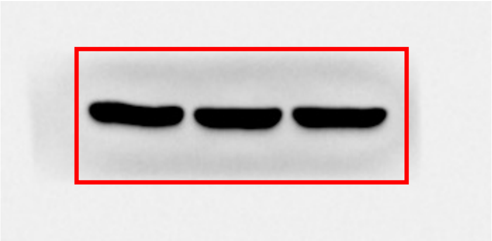

Actin (loading control of β-catenin)

# Full unedited gel for Figure S1

A

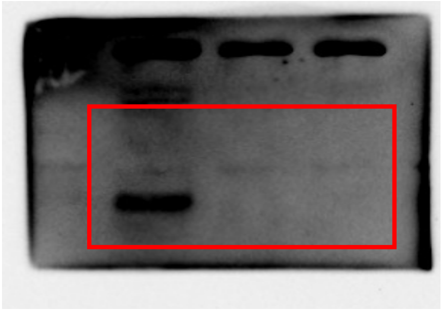


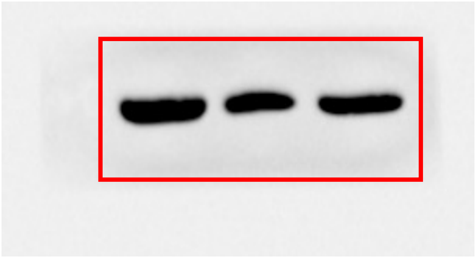


D

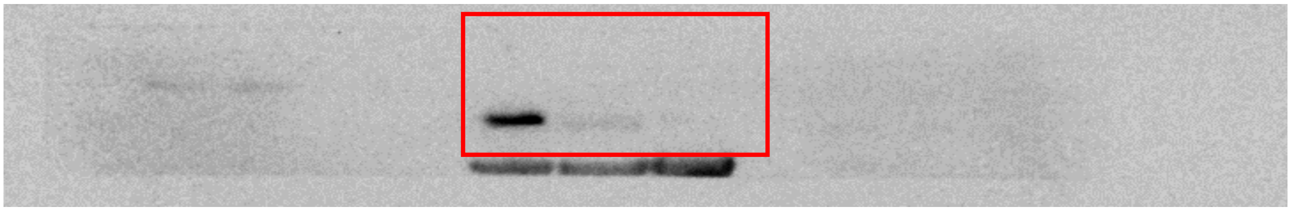


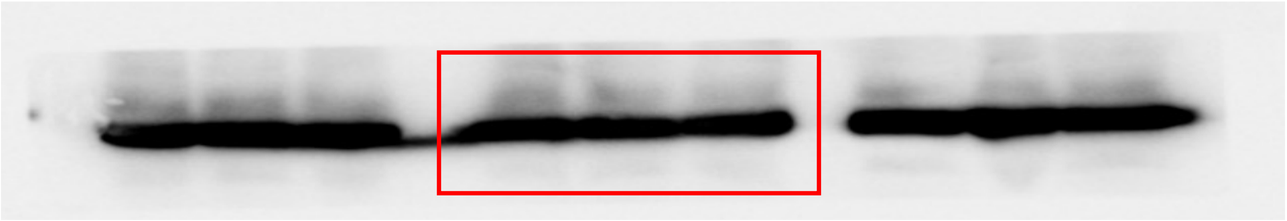

# Full unedited gel for Figure S3

A

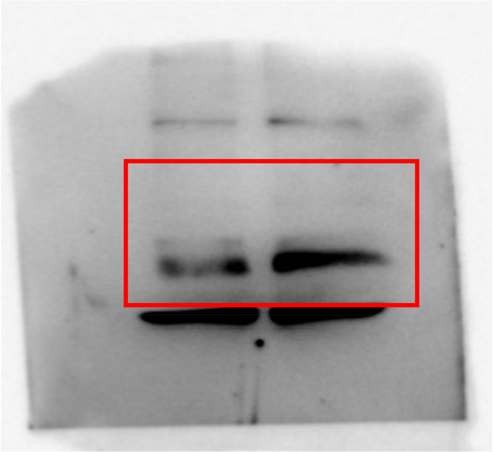

RCOR2

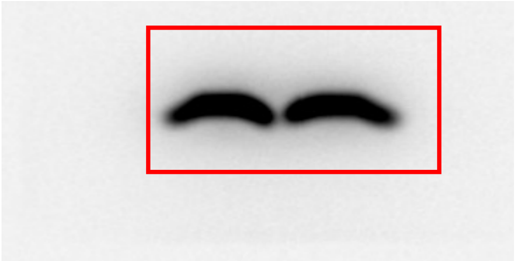

Histone H3

B

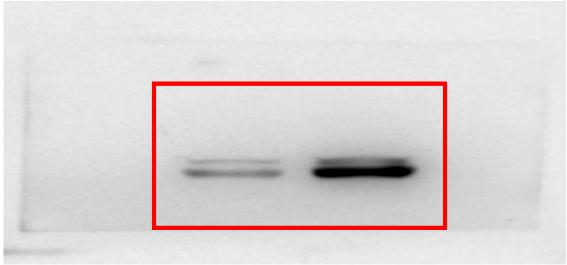

RCOR2

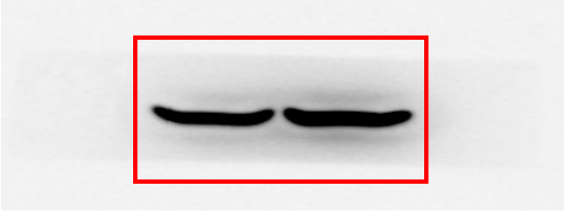

Actin

C

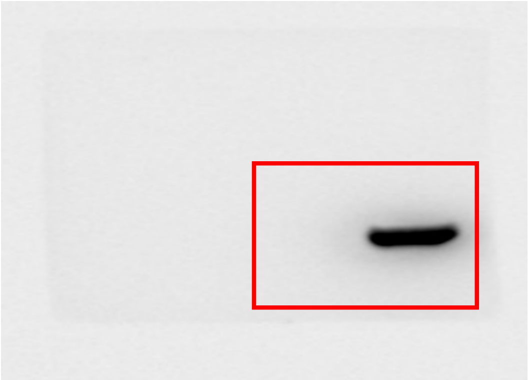

FLAG-
RCOR2

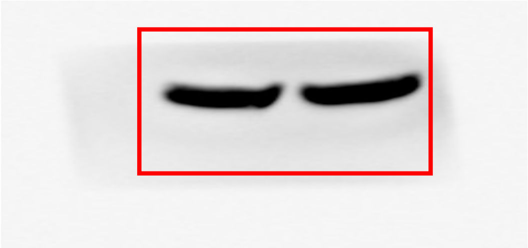

Actin

H

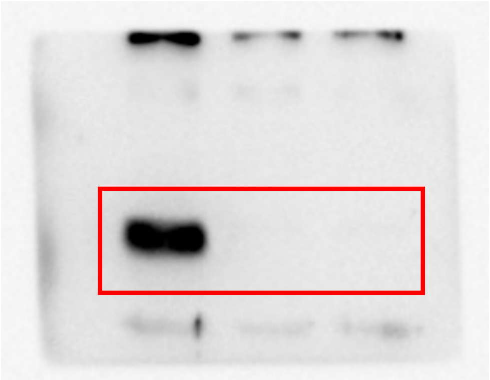

RCOR2

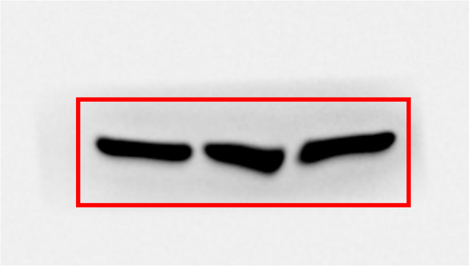

Actin

# Full unedited gel for Figure S4

C

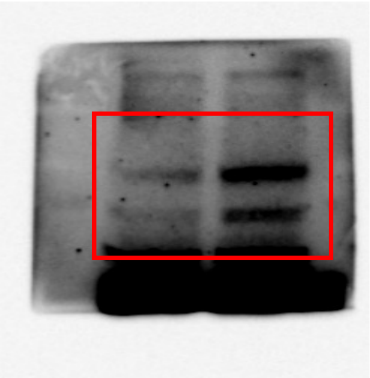

RNF43

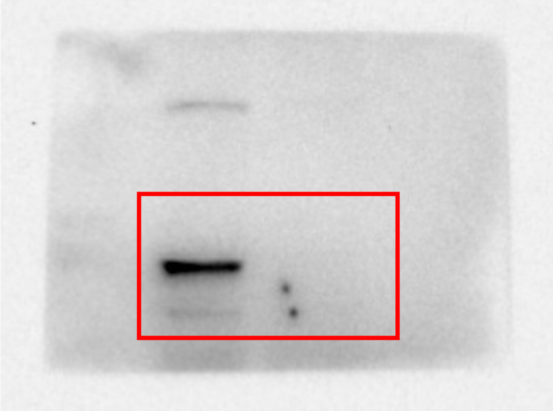

RCOR2

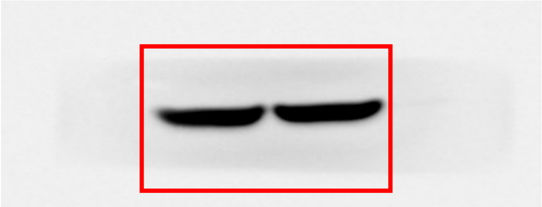

Actin

F

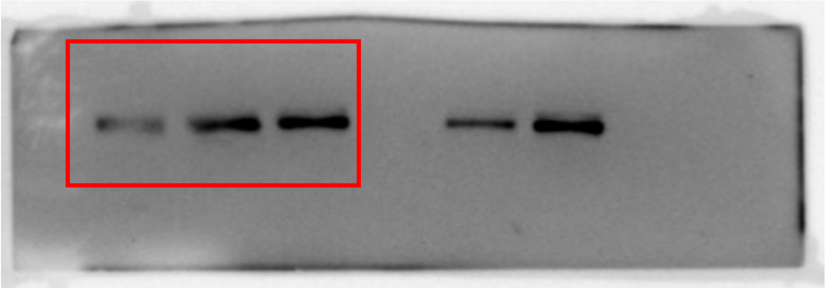

CIITA

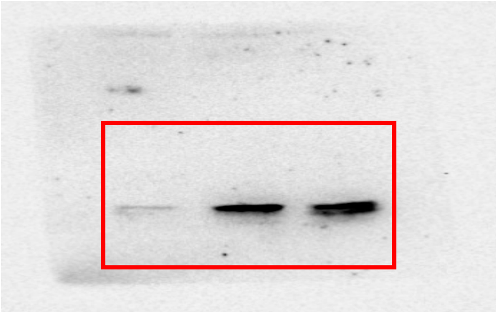

I-A/I-E

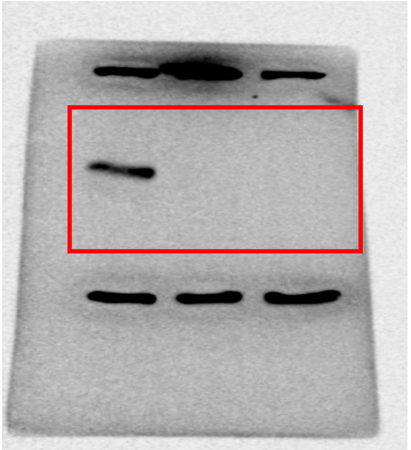

RCOR2

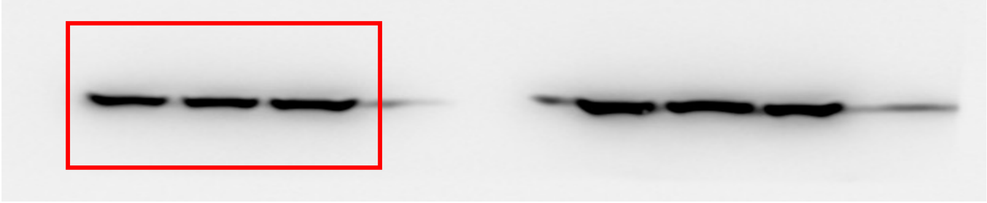

Actin

# Full unedited gel for Figure S4

H

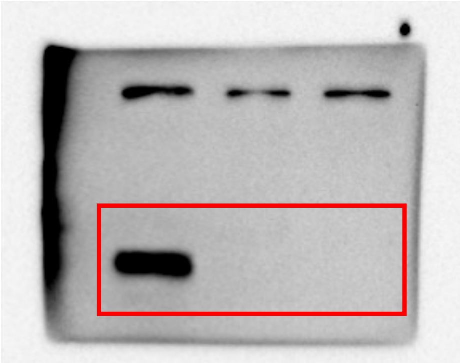

RCOR2

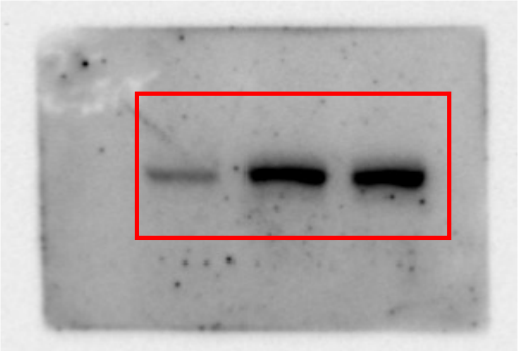

CIITA

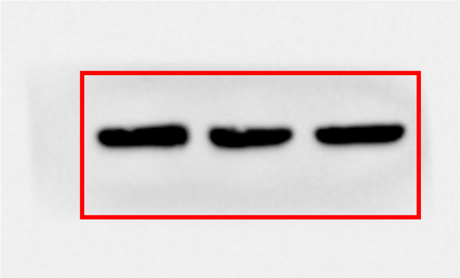

Actin
